# A Pathogen Type III Effector with a Novel E3 Ubiquitin Ligase Architecture

**DOI:** 10.1371/journal.ppat.1003121

**Published:** 2013-01-24

**Authors:** Alexander U. Singer, Sebastian Schulze, Tatiana Skarina, Xiaohui Xu, Hong Cui, Lennart Eschen-Lippold, Monique Egler, Tharan Srikumar, Brian Raught, Justin Lee, Dierk Scheel, Alexei Savchenko, Ulla Bonas

**Affiliations:** 1 Banting and Best Department for Medical Research, University of Toronto, C.H. Best Institute, Toronto, Ontario, Canada; 2 Department of Chemical Engineering and Applied Chemistry, University of Toronto, Toronto, Ontario, Canada; 3 Department of Genetics, Martin Luther University Halle-Wittenberg, Halle, Germany; 4 Leibniz Institute of Plant Biochemistry, Halle, Germany; 5 Ontario Cancer Institute and Department of Medical Biophysics, University of Toronto, MaRS TMDT 9-805, Toronto, Ontario, Canada; Ohio State University, United States of America

## Abstract

Type III effectors are virulence factors of Gram-negative bacterial pathogens delivered directly into host cells by the type III secretion nanomachine where they manipulate host cell processes such as the innate immunity and gene expression. Here, we show that the novel type III effector XopL from the model plant pathogen *Xanthomonas campestris* pv. *vesicatoria* exhibits E3 ubiquitin ligase activity *in vitro* and *in planta*, induces plant cell death and subverts plant immunity. E3 ligase activity is associated with the C-terminal region of XopL, which specifically interacts with plant E2 ubiquitin conjugating enzymes and mediates formation of predominantly K11-linked polyubiquitin chains. The crystal structure of the XopL C-terminal domain revealed a single domain with a novel fold, termed XL-box, not present in any previously characterized E3 ligase. Mutation of amino acids in the central cavity of the XL-box disrupts E3 ligase activity and prevents XopL-induced plant cell death. The lack of cysteine residues in the XL-box suggests the absence of thioester-linked ubiquitin-E3 ligase intermediates and a non-catalytic mechanism for XopL-mediated ubiquitination. The crystal structure of the N-terminal region of XopL confirmed the presence of a leucine-rich repeat (LRR) domain, which may serve as a protein-protein interaction module for ubiquitination target recognition. While the E3 ligase activity is required to provoke plant cell death, suppression of PAMP responses solely depends on the N-terminal LRR domain. Taken together, the unique structural fold of the E3 ubiquitin ligase domain within the *Xanthomonas* XopL is unprecedented and highlights the variation in bacterial pathogen effectors mimicking this eukaryote-specific activity.

## Author Summary

Numerous bacterial pathogens infecting plants, animals and humans use a common strategy of host colonization, which involves injection of specific proteins termed effectors into the host cell. Identification of effector proteins and elucidation of their individual functions is essential for our understanding of the pathogenesis process. Here, we identify a novel effector, XopL, from *Xanthomonas campestris* pv. *vesicatoria*, which causes disease in tomato and pepper plants. We show that XopL suppresses PAMP-related defense gene expression and further characterize XopL as an E3 ubiquitin ligase. This eukaryote-specific function involves attachment of ubiquitin molecule(s) to a particular protein targeted for degradation or localisation to specific cell compartments. Ubiquitination processes play a central role in cell-cycle regulation, DNA repair, cell growth and immune responses. In the case of XopL this activity triggers plant cell death. Through structural and functional analysis we demonstrate that XopL contains two distinct domains, one of which demonstrates a novel fold never previously observed in E3 ubiquitin ligases. This novel domain specifically interacts with plant ubiquitination system components. Our findings provide the first insights into the function of a previously unknown XopL effector and identify a new member of the growing family of bacterial pathogenic factors hijacking the host ubiquitination system.

## Introduction

Most Gram-negative pathogenic bacteria implement the type III secretion system (T3SS) that injects a set of proteins, termed effectors (T3E), directly into the eukaryotic host cell. The effectors' combined function is to subvert the host immune system and to promote bacterial colonization [1], [2]. Plant immunity relies on recognition of conserved pathogen-associated molecular patterns (PAMPs) [3], such as flagellin or bacterial elongation factor Tu [4], [5]. This defense barrier is termed PAMP-triggered immunity (PTI), is activated upon PAMP recognition at the cell surface by specific receptors, followed by a network of cellular signaling events, such as mitogen-activated protein kinase (MAPK) cascades, that ultimately lead to changes in gene expression [3], [6], [7]. In contrast, type III effectors manipulate plant cell processes, often leading to subversion of plant immune responses [1], [8]. T3Es interfere with key eukaryotic cell functions, such as the cytoskeleton rearrangement [9], transcriptional regulation [10], [11] or ubiquitination [12], [13]. However, the biochemical function of the majority of T3Es remains elusive.

Ubiquitination is a highly conserved eukaryote-specific post-translational protein modification involving attachment of ubiquitin to the epsilon amine of a lysine residue in the target protein. This modification alters protein activity, protein localization or targets the protein for 26S-proteasome-mediated degradation [14]. Ubiquitination of target proteins involves coupling of ubiquitin to an ubiquitin activating enzyme (E1), transfer to a conjugating enzyme (E2), before an ubiquitin ligase (E3) mediates ubiquitin transfer from an E2 to a target protein [15]. E3 enzymes exhibit high target specificity and differ in the subset of E2s they interact with. Eukaryotic E3s fall into two major classes according to the mechanism of ubiquitin transfer: RING/U-box and HECT domain proteins [16]. RING finger/U-box proteins transfer ubiquitin directly from the E2 to the target protein, whereas HECT proteins first form a thioester intermediate with ubiquitin before ligating it to the target. While ubiquitination is absent in prokaryotes, it emerges as a prime eukaryotic host target for bacterial pathogens, which have evolved diverse T3Es to mimic ubiquitination-related functions. In particular, several bacterial T3Es from animal and plant pathogens function as E3 ubiquitin ligases, represented on one hand by the *Pseudomonas syringae* T3E AvrPtoB [12], [13] and the NleG family of *E. coli* T3Es [17], which contain typical U-box folds, and on the other hand by the NEL (novel E3 ligase) domains found in the IpaH and SspH2 T3Es of *Shigella* and *Salmonella* spp., respectively [18], [19]. The latter contain a novel thioester-forming E3 ligase domain with no structural homology to the HECT domain. This suggests that during co-evolution with their hosts, pathogenic bacteria have employed different solutions to fulfill the otherwise typical eukaryote-specific function of E3 ubiquitin ligases.

Here, we characterized the T3E XopL (*Xanthomonas* outer protein L) from the model plant pathogenic bacterium *Xanthomonas campestris* pv. *vesicatoria* (*Xcv*), which causes disease on tomato and pepper plants. *Xcv* injects a suite of ∼30 T3Es into the host cell including the TAL (transcription activator-like) effector AvrBs3, which manipulates plant transcription [10], and the SUMO (small ubiquitin modifier) protease XopD [20]. XopL is a newly identified T3E from *Xcv*, and was found to exhibit E3 ubiquitin ligase activity. Crystal structure determination revealed that the protein contains a novel fold and thus represents a new class of E3 ubiquitin ligases.

## Results

### Identification of the new type III effector XopL (XCV3220)

The analysis of the genome sequence of *Xcv* strain 85-10 led to the identification of XCV3220 (*xopL*) as a new T3E candidate gene. XCV3220 is conserved in *Xanthomonas* spp. (Figure S1) and contains a PIP box (plant inducible promoter) in its promoter (TTCG-N16-TTCG; genome position 3669238-261). The presence of a PIP box in the *xopL* promoter suggested a co-regulation with the T3S system, which was confirmed by RT-PCR (Figure S2A). The predicted gene product contains leucine-rich repeats (LRRs), which are typically found in eukaryotic proteins and are thus indicative of an effector protein activity. Type III-dependent secretion and translocation of XCV3220 was confirmed by *in vitro* secretion and *in vivo* translocation assays (Figure S2B, C). The protein was therefore renamed XopL (for detailed information see Text S1).

### XopL induces cell death and suppresses defense gene expression *in planta*

**Figure 1:**
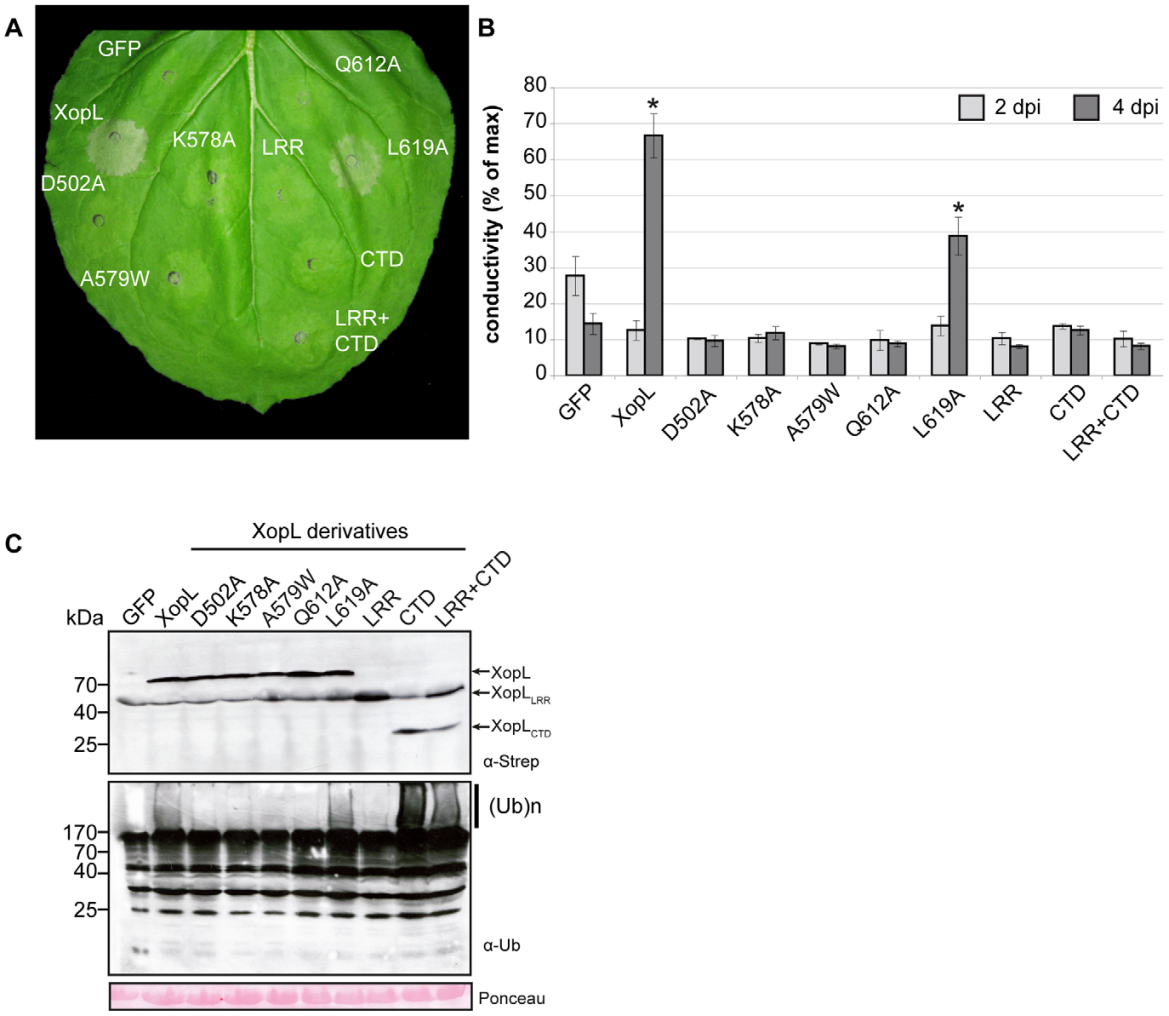
Analysis of cell death induction by XopL in *Nicotiana benthamiana*. *Agrobacterium*-strains carrying binary constructs encoding XopL (WT), XopL_D502A_ (D502A), XopL_K578A_ (K578A), XopL_A579W_ (A579W), XopL_Q612A_ (Q612A), XopL_L619A_ (L619A), XopL[aa 1–449] (leucine-rich repeat, LRR), XopL[aa 450–660] (C-terminal domain, CTD), both XopL[aa 1–449] and XopL[aa 450–660] expressed in trans (LRR+CTD) or GFP under control of the *Cauliflower mosaic virus* (CaMV) 35S promoter, were inoculated into *N. benthamiana* leaves (8×10^8^ cfu/ml). (**A**) Phenotypes of the inoculated leaf area were documented 6 days post inoculation (dpi). (**B**) Cell death quantification using electrolyte leakage measurements. Measurements were carried out 2 dpi (light grey bars) and 4 dpi (dark grey bars), respectively. Bars represent the average of triplicates of 5 leaf discs each, error bars represent standard deviations. Asterisks indicate statistically significant differences compared to GFP control (*t*-test, *P*<0.01). (**C**) Leaf tissue was harvested 2 dpi, and protein extracts were analyzsed by western blot using a *Strep*-tag (α-strep) and ubiquitin-specific antibody (α-Ub), respectively. Signals specific for full-length XopL, XopL[aa 1–449] (XopL_LRR_)and XopL[aa 450–660] (XopL_CTD_) are labeled. Polyubiquitination is indicated by (Ub)_n_. Equal loading is shown by Ponceau staining of Rubisco. The experiments were performed three times with similar results.

To investigate a possible virulence function of XopL, we deleted the gene from the genome and analyzed the corresponding deletion mutants by infection studies in pepper plants. However, under the conditions tested XopL had no discernible effect on virulence (Figure S2D) or bacterial growth of *Xcv* (data not shown). To further characterize XopL we expressed *xopL* in different plant species via *Agrobacterium*-mediated transformation. Expression of XopL induced plant cell death (PCD) in leaves of *Nicotiana benthamiana* (Figure 1A), but no macroscopic reaction in pepper or tomato plants (data not shown). PCD was confirmed by quantifying ion leakage, which is used to measure dying plant cells (Figure 1B).

**Figure 2:**
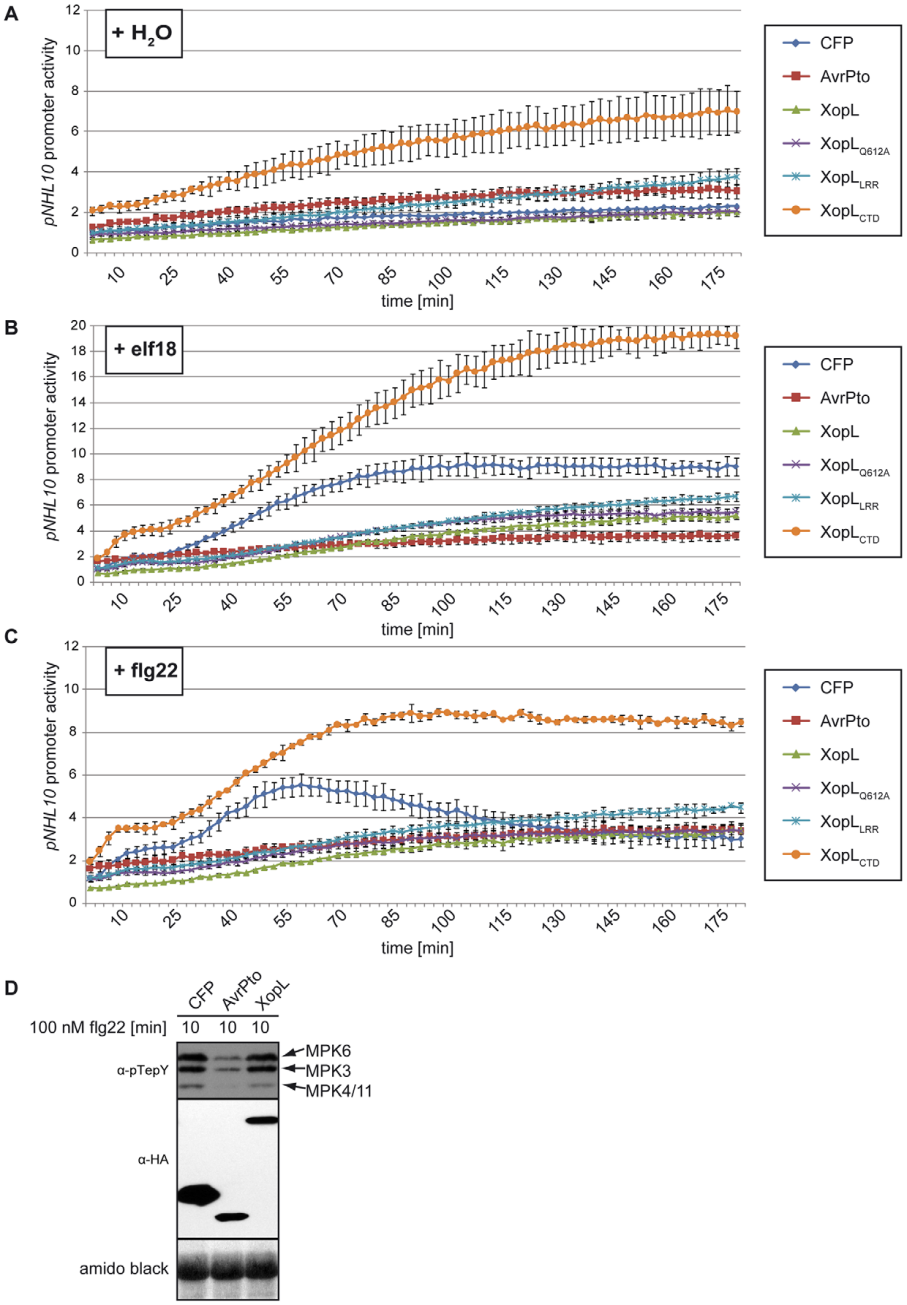
XopL inhibits pathogen-associated molecular pattern (PTI)-induced defense gene expression. *Arabidopsis thaliana* Col-0 protoplasts were co-transformed with *pNHL10-LUC* (luciferase) as reporter, the *p35S-*effector gene constructs *xopL*, *xopL_Q612A_*, *xopL_LRR_* and *xopL_CTD_* or *p35S-cfp* and p*35S*-*avrPto* (negative and positive control, respectively), and *pUBQ10-GUS* (β-glucuronidase) for normalization. 14 h after transformation, protoplasts were treated with H_2_O (**A**), 100 nM elf18 (**B**) and 100 nM flg22 (**C**), and luciferase activity was monitored for 3 h. Results are depicted as LUC/GUS ratios (with the zero timepoint, H_2_O-treated sample set at a reference value of one). (**D**) Protein extracts of transformed protoplasts were taken 10 min after treatment and analyzed by immunoblotting using a pTepY-antibody (specific for activated MAP-Kinases) and HA-specific antibodies for detection of HA-tagged effector-or CFP-fusion proteins. MPK3, 4, 6, 11: mitogen activated protein kinase 3, 4, 6, 11. The experiments were performed three times with similar results.

To identify the role of XopL during the infection of plants, we tested if it manipulates plant immunity, as shown previously for several T3Es from *Pseudomonas* and *Xanthomonas*, which specifically suppress the PAMP-triggered immunity (PTI) [21]–[26]. To analyze this, we performed *Arabidopsis* leaf protoplast assays, a well-established system for PAMP-signaling analysis [25], [27], [28]. We tested the activity of the *A. thaliana NHL10 (NDR1/HIN1-LIKE 10*) [29], [30] promoter fused to the firefly luciferase gene (*LUC*) after application of elicitor-active epitopes of different bacterial PAMPs. The reporter assays showed that the basal activity of *pNHL10* was not affected by XopL (Figure 2A). However, the expression of *xopL* significantly decreased the activation of *pNHL10* by flg22 (a bacterial flagellin epitope) [4] as well as that of elf18 (an 18 amino acid peptide derived from the EF-Tu protein) [5] (Figure 2B, C). Induction of *pNHL10* by flg22 depends, at least partially, on activity of mitogen- activated protein kinases (MAPKs) [27]. Therefore, the activation of the MAPKs MPK3, MPK4, MPK6 and MPK11, which are involved in plant immune signaling [31], [32], might be affected by XopL. However, immunoblot analysis using an antibody against activated MAPKs revealed no differences in MAPK activity in protoplasts expressing XopL (or its derivatives; data not shown) compared to CFP (cyan fluorescent protein, negative control) (Figure 2D). AvrPto served as a positive control in both assays as it suppresses PTI by intercepting MAPK signaling pathways [33]. Proteins were stably expressed and protoplasts were still viable during the course of the experiment, confirming that the lack of *pNHL10* expression was not due to ongoing cell death of the protoplasts (Figure S3A, B).

### XopL displays E3 ubiquitin ligase activity *in vitro*

The N-terminal LRRs of XopL are reminiscent of the domain architecture of the T3E families IpaH and SspH2 from *Shigella* and *Salmonella*, respectively, that were recently identified as E3 ubiquitin ligases [18], [19]. We, therefore, tested XopL for E3 ubiquitin ligase activity *in vitro*. For this, we purified recombinant full-length XopL[aa 1–660] and truncated XopL derivatives XopL[aa 144–660] (lacking the disordered pre-LRR region), XopL[aa 474–660] (lacking the LRRs) and XopL[aa 86–450] (lacking the C-terminal region).

**Figure 3:**
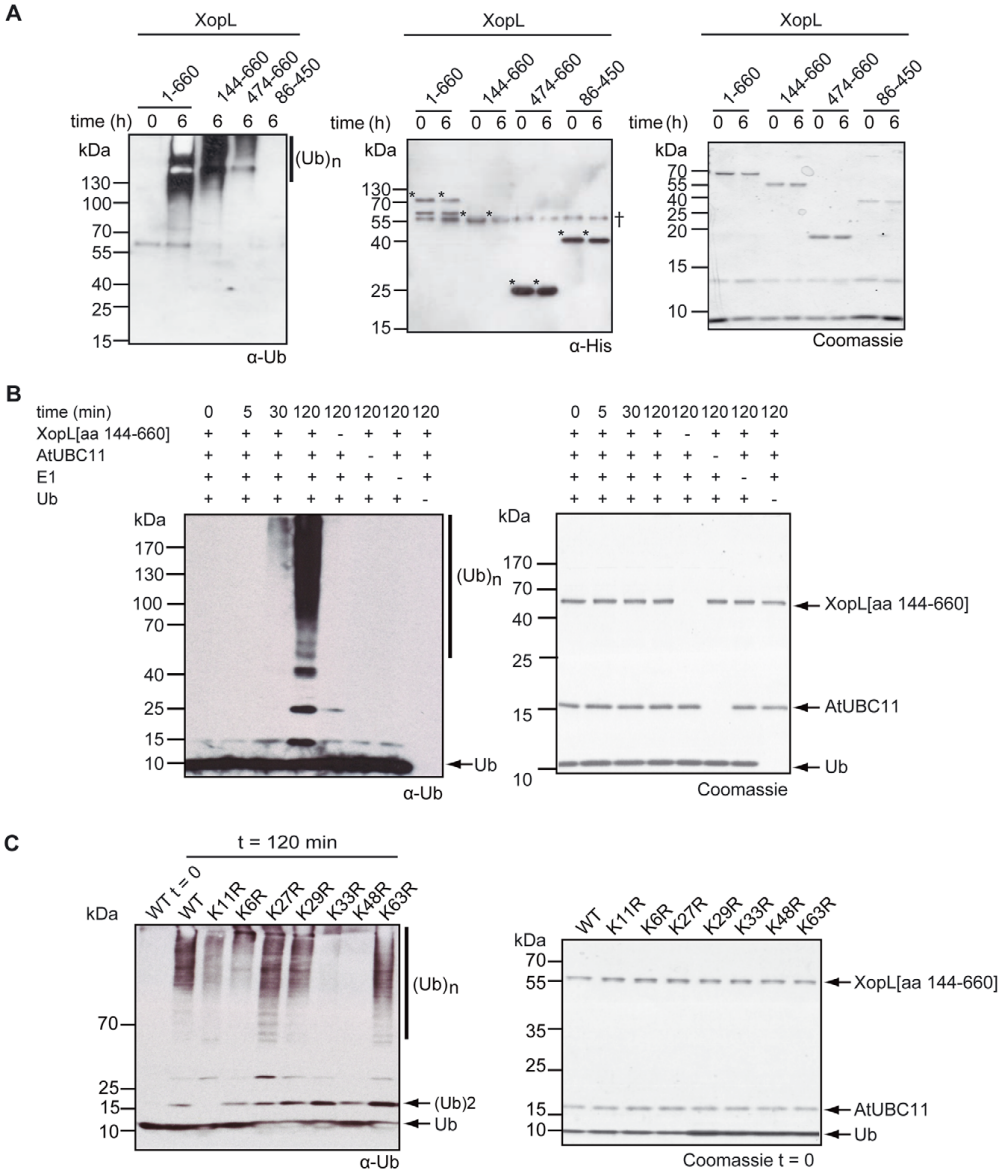
The C-terminal domain of XopL shows E3 ubiquitin ligase activity. (**A**) *In vitro* ubiquitin ligase assay in presence of E1, UBE2D2, ATP, ubiquitin and His_6_-XopL full-length protein (1–660) or derivatives thereof (numbers indicate amino acid positions corresponding to full-length protein). The western blots were reacted with antibodies against ubiquitin (α-Ub, left panel) and polyhistidine (α-His, middle panel), respectively, while the right panel shows the reaction mixture via Coomassie Blue staining of the SDS-PAGE. (Ub)_n_ indicates polyubiquitination. Asterisks indicate His_6_-XopL derivatives. Unspecific signals are labeled by †. (**B**) Ubiquitin polymerization reaction at different time points in the presence (+) or absence (−) of E1, AtUBC11 (E2), ubiquitin and His_6_-XopL[aa 144–660]. Polyubiquitination was determined by western blot (left panel) using ubiquitin antibodies. The right panel shows the state of modification of the proteins via Coomassie Blue staining of the 10–15% step-gradient SDS-PAGE gel. Components of the reactions (XopL[aa 144–660], ubiquitin (Ub) and AtUBC11) on western blots or Coomassie-stained gels are labeled. (**C**) *In vitro* ubiquitination assay in the presence of ATP, E1, AtUBC11, His_6_-XopL[aa 474–660], ubiquitin (WT) and lysine (K) to arginine (R) mutant derivatives thereof. The left panel shows the western blots probed against ubiquitin (α-Ub) of the *in vitro* reactions, run on a 10–15% step-gradient SDS-PAGE using ubiquitin mutant derivatives in which only the indicated K residues were substituted by R. The right panel shows the starting material of the *in vitro* reactions in the left panel via Coomassie Blue staining of the SDS-PAGE. Polyubiquitination is indicated by (Ub)_n_; the components of the reactions and the di-ubiquitin (Ub)2 on the western blot or Coomassie-stained SDS-PAGE are labeled.

**Figure 4:**
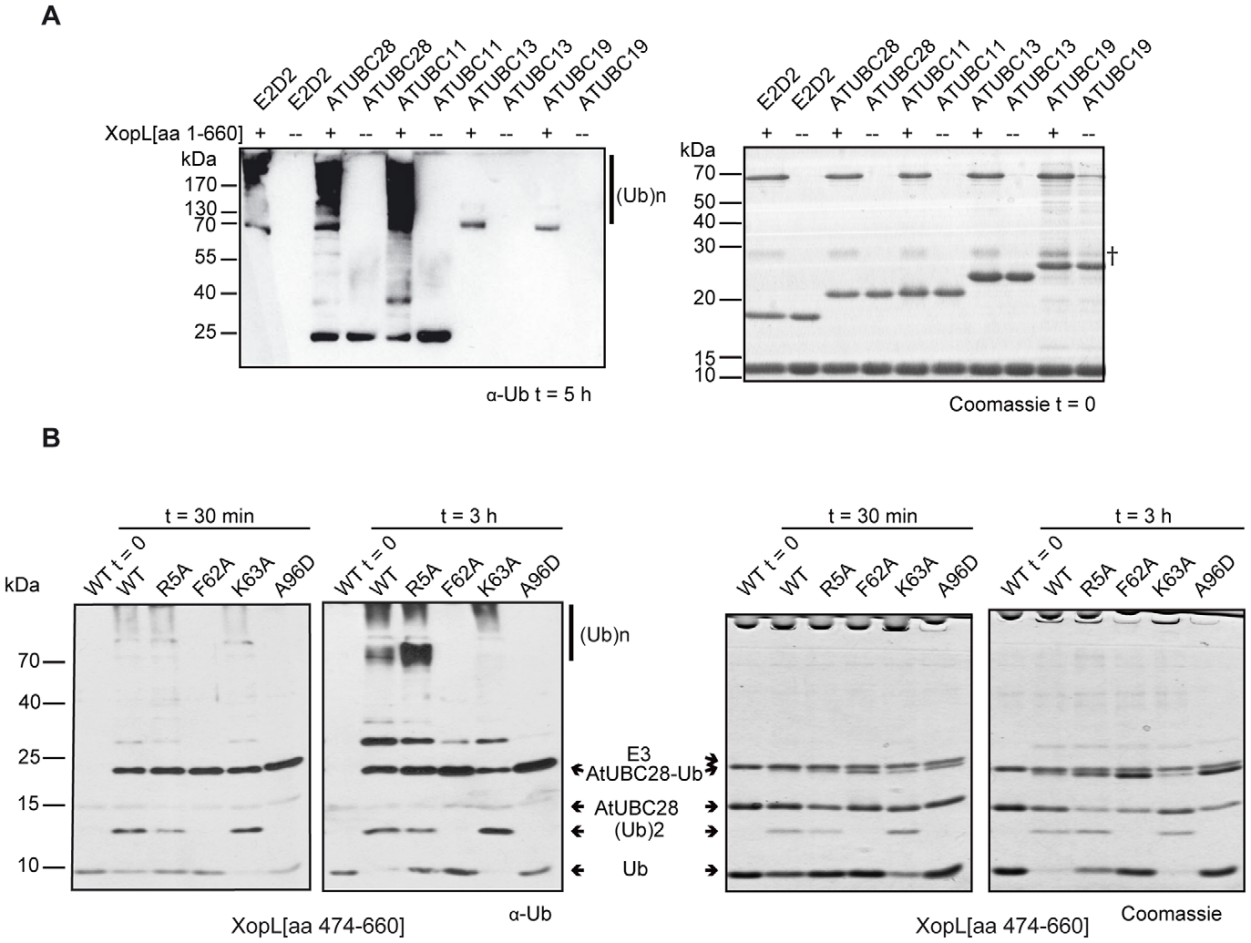
XopL displays E2 specificity *in vitro*. (**A**) *In vitro* ubiquitin ligase assay with ATP, ubiquitin, E1, human UBE2D2 (E2D2) or different *Arabidopsis thaliana* E2s (ATUBC28, 11, 13 or 19) in the presence (+) or absence (−) of His_6_-XopL[aa 1–660]. The left panel shows the western blot reacted with ubiquitin antibodies (α-Ub) after 5 hours incubation, while the right panel shows the Coomassie stained gel of the reactants at the start of the reaction. Polyubiquitination is indicated by (Ub)_n_. A lower-molecular weight impurity or degradation product in the full-length XopL protein purification is denoted by †. (**B**) Ubiquitin ligase assay described in (A) using His_6_-XopL[aa 474–660], AtUBC28 and mutant derivatives R5A, F62A, K63A and A96D. Reaction times are indicated. The left panel shows the western blot reacted with ubiquitin antibodies (α-Ub), while the right panel shows the Coomassie-stained gel at the equivalent time points. (Ub)_n_ indicates polyubiquitination, and positions on the western blot or Coomassie-stained gels corresponding to ubiquitin (Ub), di-ubiquitin (Ub)2, AtUBC28, mono-ubiquitinated AtUBC28 (AtUBC28-Ub) and His_6_-XopL[aa 474–660] (E3) are labeled.

XopL and its derivatives were tested in ubiquitination assays using human E1 and the ubiquitous human E2 (UBE2D2) or the related plant E2s (AtUBC11 or AtUBC28, both with ∼80% sequence identity to UBE2D2) enzymes. In the case of full-length XopL, XopL[aa 144–660] and XopL[aa 474–660], western blot analysis with ubiquitin antibodies revealed a robust time-dependent accumulation of high-molecular-weight polyubiquitinated protein species (Figure 3A, B), which at later time points correlated with consumption of free ubiquitin (Figure 4B). A similar result was also obtained for the more distantly related XopL from *X. c.* pv. *campestris* (Table S1 in Text S1).

Western blot analysis using α-His antibodies (Figure 3A) and Coomassie Blue staining of SDS-PAGE gels (Figure 3B), combined with mass spectrometric analysis of the high-molecular weight species (data not shown) demonstrated minimal modification of the XopL fragments, indicating that the principle product of *in vitro* ubiquitination reactions were unattached ubiquitin chains. In the case of the XopL[aa 86–450] fragment, no polyubiquitinated protein species were detected (Figure 3A), suggesting that polyubiquitination was dependent on the intact XopL C-terminal region. XopL-mediated formation of ubiquitin chains required both E1 and E2 enzymes (Figure 3B), demonstrating that XopL acts similarly to eukaryotic E3 ubiquitin ligases.

Next, we determined the type of ubiquitin linkages preferentially generated by XopL. Ubiquitin contains seven lysine (K) residues (K6, K11, K27, K29, K33, K48 and K63) that can participate in ubiquitin ligation [14]. Therefore, we analyzed the products of the XopL-mediated polyubiquitination reaction using plant AtUBC11, AtUBC28 and human UBE2D2 conjugating enzymes. While the relative amount of distinct ubiquitination linkages detected by this analysis (Table S1 in Text S1) was different depending on which E2 enzyme was used in the reaction, the K11 linkages represented the largest fraction in all cases. More than half of the linkages analyzed in reactions with AtUBC28 and UBE2D2 enzymes were K11, whereas K11 represented ∼45% of the linkages in reactions with AtUBC11. The remaining polyubuitination linkages corresponded primarily to K33, K48 and K63 (Table S1 in Text S1). Interestingly, K63-linked polyubiquitin chains were detected in reactions using AtUBC28 and human UBE2D2 but not in reactions with plant AtUBC11, suggesting that these homologous E2 enzymes may contribute to a different preference in linkages that are formed during E3 catalyzed reactions.

In order to confirm the prevalence of the detected linkages in the XopL-mediated reaction we then performed polyubiquitination assays using ubiquitin variants with each individual lysine residue mutated to arginine (Figure 3C). In accordance with mass spectrometry results, the K11R mutation significantly dampened the XopL-mediated formation of polyubiquitin chains in the reaction using the AtUBC11 enzyme. A similar effect was detected in case of K33R and K48R mutations. Interestingly, the K6R mutation also resulted in significant reduction of polyubiquitination, while no K6 linkages were detected among XopL polyubiquitination products. This result suggested that this mutation might have a general deleterious effect on ubiquitination, potentially due to reduced affinity to E1 or E2 enzymes.

### XopL interacts with specific plant E2 enzymes

Next, we tested XopL ubiquitin ligase activity with different plant-derived E2s. As stated above, XopL forms ubiquitin chains with AtUBC11 and AtUBC28 (93% sequence identity), which belong to group VI of the 16 E2 classes of this plant [34], and the close human homologue UBE2D2. However, two more distantly related E2s (Table S1 in Text S1), namely AtUBC13 (group V, 34% sequence identity to AtUBC11) and AtUBC19 (group VIII, 43% sequence identity to AtUBC11) did not show any activity in our *in vitro* assays (Figure 4A), suggesting that XopL discriminates between different classes of E2 enzymes, as was described for other E3 ubiquitin ligases [19], [35]–[37].

Interactions between the human UBE2D2 enzyme and E3 ubiquitin ligases have been studied in detail by mutagenesis [38]. Because mutation of conserved residues in UBE2D2 abrogated ubiquitination *in vitro*, we purified the R5A, F62A, K63A and A96D variants of the AtUBC28 E2 enzyme and tested them individually in XopL and XopL[aa 474–660] ubiquitination assays.

The F62A and A96D mutations in AtUBC28 completely abrogated both the XopL[aa 474–660]- and XopL-mediated polyubiquitination reactions (Figure 4B; data not shown), suggesting that F62 and A96 are required for the AtUBC28 interaction with XopL. By contrast, the AtUBC28 R5A and K63A mutants were still very active *in vitro* (Figure 4B).

Taken together, our results demonstrate that XopL is an E3 ubiquitin ligase that selectively recruits plant E2 enzymes.

### Structural analysis of the XopL N- and C-terminal domains reveals a novel fold

The XopL C-terminal domain harboring E3 ubiquitin ligase activity lacks significant sequence similarity with previously characterized E3 ligases. To gain further insight into the structural basis of XopL activity, we determined the structure of XopL by X-ray crystallography. While full-length XopL did not crystallize, fragments XopL[aa 144–450] and XopL[aa 474–660] yielded crystals that diffracted to a resolution of 2 Å and 1.8 Å, respectively. In both cases, single-wavelength anomalous dispersion (SAD) data were collected at the selenium peak wavelength from a single selenomethionine-enriched crystal. The final model of XopL[aa 144–450] contained a single molecule in the asymmetric unit corresponding to residues 145 to 437 plus four additional residues from the N-terminal polyhistidine tag. For the XopL[aa 474–660] fragment, three polypeptide chains were found in the asymmetric unit corresponding to residues 474–642 plus up to six residues from the N-terminal polyhistidine tag. Data collection and refinement statistics for both structures are presented in Table 1.

**Table 1:** Data collection, phasing and refinement statistics for SAD (SeMet) structures.

	XopL[aa 144–450]	XopL[aa 474–660]
**Data collection**		
Space group	*C2221*	*P32*
Cell dimensions		
*a*, *b*, *c* (Å)	50.5, 95.2, 115.5	119.2, 38.7
α, β, γ (°)	90, 90, 90	90, 90, 120
	*Peak*	*Peak*
Wavelength	0.97937	0.97921
Resolution (Å)	50-2.00 (2.03-2.00)*	100-1.8(1.83-1.80)
*R*_sym_ or *R*_merge_ a	0.072(0.364)	0.063(0.519)
*I*/σ*I*	38.4(4.9)	25.0(1.85)
Completeness (%)	99.9(99.3)	99.4(99.0)
Redundancy	7.3(5.4)	3.2(2.6)
**Refinement**		
Resolution (Å)	29.9-2.00	28.6-1.80
No. reflections	19119	110283
*R*_work_ b/*R*_free_ c	17.1/22.6	15.2/19.6
No. atoms	2491	4542
Protein	2400	4119
Ligand/ion	23	14
Water	168	409
*B*-factors		
Protein	30.7	34.3
Ligand/ion	47.3	40.4
Water	37.2	37.0
R.m.s deviations		
Bond lengths (Å)	0.007	0.004
Bond angles (°)	1.1	0.76

* Values in parentheses are for highest-resolution shell.

a *R*_merge_ = Σ|*I*−<*I*>|/Σ *I*.

b *R*_work_ = Σ | *F*_obs_−*F*_calc_ |/Σ |*F*_obs_|, where *F*_obs_ and *F*_calc_ are observed and calculated structure factors, respectively.

c *R*_free_ calculated using 5% of total reflections randomly chosen and excluded from the refinement.

**Figure 5:**
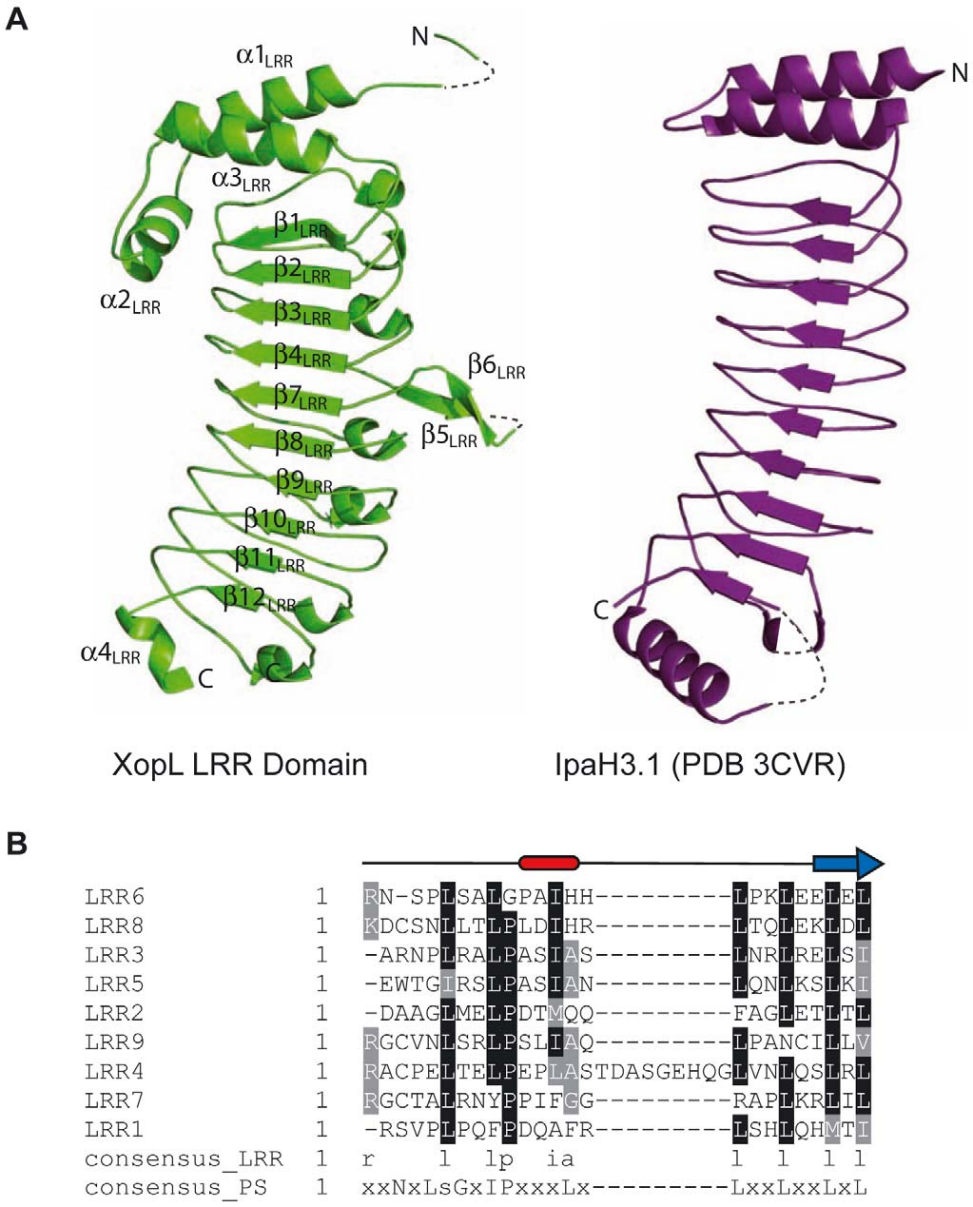
Structure of the N-terminal LRR domain of XopL. (**A**) The left panel shows the ribbon diagram of the XopL[aa 144–450] structure (green). N- and C-termini and the secondary structure elements (see Figure S1) are labeled. In comparison, the IpaH3 LRR domain (PDB code 3CVR), represented by aa residues 25–268, is shown in the right panel as a ribbon diagram (purple) with labeled N- and C-termini. Disordered regions in the protein are represented as grey dashed lines. (**B**) Sequence alignment of the nine leucine-rich repeats of XopL[aa 145–450], showing their consensus and relationship to the plant-specific (PS)-LRR subclass of LRRs. The positions of the helical turn (red box) and β- strand (blue arrow) in the “typical” LRR of XopL are given.

The structure of the XopL[aa 144–450] fragment follows a canonical LRR architecture with ten β-strands and nine complete repeats each folding into an α-helix (single turn)-turn-β-strand motif (Figure 5A). Three α-helices (α1, α2 and α3) and one α-helix (α 4) cap the LRRs at the N- and C-terminus, respectively. This structure is similar to the LRR domain of IpaH3 (PDB 3CVR [39], Figure 5A). Based on the sequence conservation at specific positions in individual repeats, a consensus sequence for the XopL LRRs can be derived that is similar to that of plant derived LRR-containing proteins (Figure 5B).

**Figure 6:**
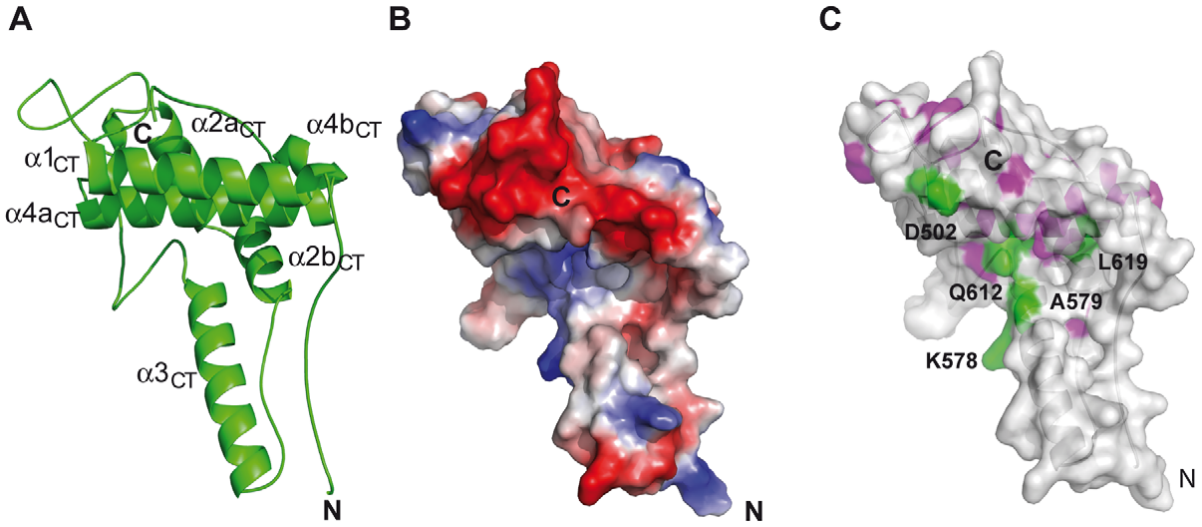
Structure of the XL-box of XopL. (**A**) Ribbon diagram of a single molecule (molecule B) of the 3 molecules in the asymmetric unit of the XopL[aa 474–660] structure. Secondary structure elements (according to the nomenclature in Figure S1) and the N- and C-termini are labeled. (**B**) Electrostatic surface of molecule B from the same structure, using the same view. Electrostatic potential was calculated using the default values from PYMOL (http://www.pymol.org/). (**C**) Same surface as in (B), showing the absolutely conserved residues from the alignment in Figure S1 (colored pink on the semi-transparent surface ). The surface is semi-transparent showing a ribbon representation of the structure. Residues absolutely conserved and subject to mutation are colored green and labeled.

The structure of the XopL C-terminal region [aa 474–660] represents a four-helix bundle, which can be subdivided into two uneven lobes almost perpendicular to each other (Figure 6A). The smaller lobe contains the N-terminus, α2b and α3 helices and a region C-terminal to the α2b helix (residues 554–562), which adopts a conformation intermediate between a poly-proline type II helix and a β-strand. The two lobes give the XopL[aa 474–660] molecule an “L”-shape, and a large cleft with a net negative charge is formed at the intersection of the two lobes (Figure 6B, C). A search for structural homology using the DALI server (http://ekhidna.biocenter.helsinki.fi/dali_server/, 2012) did not reveal any significant similarity between the XopL[aa 474–660] structure and other structurally characterized proteins including E3 ubiquitin ligases. This analysis clearly demonstrates that the XopL C-terminal domain represents a novel fold, which we termed XL-box (XopL E3 ligase box). The XL-box lacks cysteine residues. Therefore, XopL E3 ubiquitin ligase activity appears not to involve the formation of thioester intermediates with ubiquitin as was shown in the case of eukaryotic (HECT-type) and effector (IpaH and SopA) catalytic E3 ubiquitin ligases.

### The LRR and XL-box domains play different roles *in planta*

Given that structural analysis defined the presence of two distinct domains in XopL (LRR and XL-box), we tested their individual role in suppressing PAMP-induced gene expression and inducing PCD (see above; Figure 1A). When the N-terminal [aa 1–449] and the C-terminal [aa 450–660] XopL regions were expressed individually or co-expressed in *N. benthamiana*, no PCD was observed (Figure 1A, B) demonstrating that an intact XopL protein is required to provoke PCD, which is consistent with the suggested function of the LRRs in recognition of a plant target protein ubiquitinated by the XL-box. Next, we tested the effect of mutations in the XL-box domain on the ability of XopL to provoke PCD (Figure 1A, B; Figure S5A, B; Table S3 in Text S1). Residues D502, K578, A579, Q612 and L619 co-localize on the surface of the major cleft of the XL-box (Figure 6C), and are highly conserved (Figure S1). Each of the aforementioned residues was substituted by alanine, except for A579, which was mutated to tryptophan. Transient expression of these XopL variants in *N. benthamiana* revealed that the XopL mutant derivatives were stably synthesized (Figure 1C) and D502A, K578A, A579W or Q612A exchanges abolished the ability of XopL to induce PCD. By contrast, the XopL_L619A_ variant was still active (Figure 1A, B). We then investigated if E3 ubiquitin ligase activity of XopL can be demonstrated in the plant. *N. benthamiana* leaves expressing full-length XopL, XopL[aa 1–450], XopL[aa 450–660] or GFP (green fluorescent protein; control) were analyzed by western blotting using ubiquitin-specific antibodies. Expression of full-length XopL and XopL[aa 450–660], but not XopL[aa 1–450] led to the presence of additional high molecular mass ubiquitinated protein species, that were not detected upon expression of *gfp* (Figure 1C).

Notably, the D502A, K578A, A579W and Q612A mutations that abrogated the ability of XopL to cause PCD also dampened the formation of polyubiquitin chains *in vivo* (Figure 1C, Figure S5C). On the other hand, XopL_L619A_ caused PCD and retained the ability to mediate formation of polyubiquitin chains *in vivo* similarly to the wild type. A similar result was found performing *in vitro* polyubiquitination reactions using the AtUBC11 conjugating enzyme and XopL[aa 474–660] (Figure S6). Taken together these results suggested that PCD is caused by XopL E3 ligase activity, manifested by formation of polyubiquitin products *in vivo* and *in vitro*.

We also tested the effect of the individual domains on suppression of PAMP-induced gene expression relative to full-length XopL (Figure 2, Figure S3). Unexpectedly, the PAMP-suppression activities of XopL are mediated by the N-terminal (residues 1–450) fragment corresponding to the LRR-containing region, which suppressed PAMP-induced gene expression to a similar extent as the full-length XopL. In addition, full-length XopL with a Q612A mutation in the XL-box, which both strongly hinders the ability of XopL to promote PCD and to polyubiquitinate *in vivo* and *in vitro*, retained the ability to inhibit the expression of the reporter gene in the presence of either PAMP elicitor peptides. Finally, the expression of the XopL_CTD_ did not suppress, but rather elevated, the expression of the reporter even in the absence of the PAMP elicitor peptides (Figure 2A–C).

## Discussion

In this study, we identified XopL as a new T3E in *Xcv* that induces cell death in *N. benthamiana* and inhibits PTI-related defense gene expression. According to our data, XopL exhibits a robust E3 ubiquitin ligase activity. This activity is associated with its C-terminal region and is required for induction of plant cell death. All ubiquitin ligases known to date including bacterial T3Es with E3 ligase activity belong to the RING/U-box or catalytic (HECT-like) class [16]. RING/U-box proteins act by transferring ubiquitin from E2 directly onto the target protein. T3Es of this class include AvrPtoB from the plant pathogen *P. syringae* [13], and *E. coli* NleG [17]. Both T3Es lack significant sequence similarity with RING/U-box proteins but adapt a protein fold similar to that of U-box proteins. On the other hand, the catalytic HECT E3 ligases first attach ubiquitin from the E2 to a catalytic cysteine residue via a thioester intermediate before ligating it to the target protein. A similar mechanism has been adopted by effector proteins of the IpaH and SopA families of animal pathogens [40], [41]. The IpaH and SopA crystal structures are distinct from HECT proteins except for the presence of a catalytic cysteine and certain features of the active site. As XopL lacks cysteine residues in its C-terminal domain, termed XL-box, we hypothesize that it acts by directly transferring ubiquitin from E2 onto a target protein. This is reminiscent of RING/U-box proteins; however, XopL lacks any structural similarity to these E3 ligases.

We found that XopL interacts *in vitro* through its XL-box with a specific family of E2 enzymes, represented by human UBE2D2 and *Arabidopsis* AtUBC11 and AtUBC28. In *Arabidopsis thaliana*, AtUBC11 and AtUBC28 are members of the group VI family of E2 enzymes [34]. Many of the 8 family members are ubiquitously expressed in *Arabidopsis* (including AtUBC28 and AtUBC11) and the three most highly expressed members of this family (AtUBC8, AtUBC10 and AtUBC28; www.genevestigator.com) share 97% sequence similarity with each other. Homologues to these proteins are also found in tomato (*S. lycopersicum* gi|350536447|; 97% identical to AtUBC28) and pepper (*C. annuum* gi|40287554|; 96% identical to AtUBC28). Mutation analyses of AtUBC28 revealed amino acid residues F62 and A96 to be critical for the interaction with the XopL E3 ligase. It is worth noting that residue F62 is essential for E2 interactions with HECT E3 ligases [42], but not for interactions with specific RING/U-box proteins [43]. On the other hand, residue A96 in E2 enzymes was shown to contribute to interactions with both HECT- and RING-type ligases plus the bacterial effector SspH2 [44]. While this data reveals some molecular details of the XopL interaction with E2 enzymes it cannot be modeled according to previously characterized E3-E2 pairs and requires further structural analysis.

XopL-mediated polyubiquitin chains with preponderance of K11 linkages were detected using both *Arabidopsis* group VI E2 enzymes and the human UBE2D2 enzyme. Ubiquitin contains seven lysine residues that can participate in target protein ubiquitination. Which specific lysine is used is dictated by different E3-E2 enzyme combinations and may trigger different outcomes for a given target protein. Linkage at K48 usually directs target proteins to the proteasome [45], whereas K63-ubiquitination can play a role in signal transduction [46]. The importance of other ubiquitin linkages for cell processes came to light only recently and their physiological role remain largely unknown [47]. A recent report suggested that mixed K11- and K63-linked chains are a virus-internalization signal [48]. In addition, K11-linked ubiquitin chains have been connected to degradation of substrates of the anaphase-promoting complex in cell cycle regulation [49], [50]. The *Salmonella* T3E E3 ubiquitin ligase SspH2, which similarly to XopL selectively interacts with the human UBE2D2 enzyme, mediates the formation of primarily K48-linked polyubiquitin chains [44]. Considering the predominance of K11-linked polyubiquitination in the case of the interaction between XopL and UBE2D2 or its plant homolog we speculate that K11-linked ubiquitin chains may play an important role in plant-pathogen interactions. However, this remains to be elucidated.

Our structural data confirmed that XopL harbors a *bona fide* LRR domain. The LRR domain is a common feature between XopL and the IpaH- and SspH2- effector E3 ubiquitin ligases mentioned above. While the LRR domain in IpaH plays a regulatory role by inhibiting the E3 activity in the absence of the substrate [18], [19], [51], there is no indication for this kind of mechanism in the case of XopL, as E3 ligase activity is robust in the presence or absence of the LRR domain. However, we were surprised to find that the LRR is involved in suppression of PAMP-elicited gene expression, which we performed using the well-established Arabidopsis protoplast system. According to our data the expression of *pNHL10* following elicitation of protoplasts with either flg22 or elf18 peptides was suppressed by the LRR domain, similarly to full-length XopL. This argues for an adaptor function of the LRR domain in which the LRR domain binds a target downstream of PAMP-receptor binding and either downstream or independent of MAPK cascade-signaling, leading to altered gene expression. These results are reminiscent to those reported for the *Pseudomonas* type III effector AvrPtoB, where suppression of plant immunity by blocking downstream signaling through BAK1-kinase are due exclusively to the two binding domains localized to residues 121–205 and 270–359 [52].

As shown by our *in planta* ubiquitination profiles, the presence of both the LRR and XL-box domains is essential for XopL-induced reactions. While expression of the XL-box domain *in planta* resulted in formation of additional polyubiquitin chains, in line with its *in vitro* activity, only full-length XopL with an intact LRR domain triggered cell death. In addition, expression of the individual XL-box and LRR domain had the opposite effect on expression of the *NHL10* promoter, even in the absence of PAMP-response elicitor. This suggests that the LRR domain functions as a protein-protein interaction module necessary for both the suppression of PAMP-elicited gene expression and the cell death phenotype we observed. Thus, we hypothesize that XopL fulfills multiple functions *in planta* by (i) suppressing PTI via its LRR-region and (ii) ubiquitinating a yet unknown plant substrate(s) whose initial recognition may also require the LRR-region.

In conclusion, characterization of the bacterial pathogen effector XopL uncovered a novel E3 ubiquitin ligase fold that is part of the pathogen repertoire to mimic an otherwise strictly eukaryotic function such as ubiquitination. This underlines the variety of E3 ligases evolved in pathogenic bacteria for subverting host biology. The next challenge is the identification of host targets of XopL that are involved in suppression of plant defenses, as well as determination of the mechanism of action of this unusual E3 ligase.

## Materials and Methods

### Bacterial strains and growth conditions

*Escherichia coli* cells were cultivated in lysogeny broth medium (LB) at 37°C. *Agrobacterium tumefaciens* was grown at 30°C in YEB (yeast extract broth) medium and *Xcv* at 30°C in NYG (nutrient yeast glycerol, [53]) or secretion medium (minimal medium A, [54]) supplemented with 10 mM sucrose and 0.3% casamino acids. Plasmids were introduced into *E. coli* and *A. tumefaciens* by electroporation and into *Xcv* by conjugation, using helper plasmid pRK2013 in triparental matings [55].

### Plant material and inoculations

The near-isogenic pepper (*Capsicum annuum*) cultivars ECW, ECW-10R and ECW-30R [56] were grown at 23°C with 60% relative humidity and 16 h light and *Nicotiana benthamiana* plants were grown at 22°C with 60% relative humidity and 16 h light. *Xcv* strains were inoculated with a needleless syringe into leaves at 10^8^ colony-forming units (cfu)/ml in 10 mM MgCl_2_. For *in planta* transient expression studies, *A. tumefaciens* strain GV3101 [57] was incubated in inoculation medium (10 mM MgCl_2_, 5 mM MES, pH 5.3, 150 µM acetosyringone) and inoculated into leaves at 8×10^8^ cfu/ml.

### Protein analysis

*Xanthomonas in vitro* secretion experiments were performed as described [58]. Equal amounts of total bacterial cell extracts and culture supernatants were analyzed by SDS-polyacrylamide gel electrophoresis (PAGE) and immunoblotting following standard protocols. To exclude bacterial lysis, blots were routinely reacted with an antibody specific for the inner membrane lipoprotein HrcJ [59]. To analyze *Agrobacterium*-mediated protein expression, two leaf discs (0.9 cm in diameter) were frozen and ground in liquid nitrogen, resuspended in 100 µl 8 M urea and 50 µl 5× Laemmli buffer, and boiled for 10 min. Proteins were separated by SDS-PAGE and analyzed by immunoblotting. We used polyclonal antibodies for detection of AvrBs3 [60] and ubiquitin (Abcam, Cambridge, U.K.), and a monoclonal *Strep*-tag antibody (IBA GmbH, Göttingen, Germany). Horseradish peroxidase-labeled α-rabbit and α-mouse antibodies (Amersham Pharmacia Biotech, Piscataway, N.J., U.S.A.) were used as secondary antibodies. Antibody reactions were visualized by enhanced chemiluminescence (Amersham Pharmacia Biotech).

### RNA analysis

RNA extraction from *Xanthomonas*, cDNA synthesis and reverse transcription polymerase chain reaction (RT-PCR) experiments were performed as described [61].

### Generation of a *xopL* deletion strain

To generate a genomic deletion of *xopL* 2 kb and 1.1 kb fragments upstream and downstream of *xopL* were amplified by PCR from genomic DNA of *Xcv* 85-10 using oligonucleotides harboring appropriate restriction sites. PCR-fragments were cloned into the suicide vector pK18mobsac [62]. The resulting constructs were conjugated into *Xcv* strain 85-10 and *xopL* deletion mutants were selected by PCR.

### *xopL* expression constructs

To generate binary expression constructs, the coding sequence of *xopL* was amplified by PCR, fused to a *Strep*-tag-coding sequence, cloned into pENTR/D-TOPO (Invitrogen GmbH, Karlsruhe, Germany) and recombined into pGWB2 [63] using GATEWAY technology (Invitrogen). XopL-derivatives listed in Table S3 (in Text S1) were generated using the Phusion Site-Directed Mutagenesis Kit (Fisher Scientific GmbH, Schwerte, Germany). To generate *avrBs3Δ2*-fusions, the promoter and 5′coding sequence of *xopL* were amplified by PCR, cloned into pENTR/D-TOPO and recombined into pL6GW356 [64]. Sequences of oligonucleotides are available upon request.

### Electrolyte leakage measurements and statistical analysis

Triplicates of five leaf discs each (0.9 cm in diameter) were harvested 2 dpi and 4 dpi. Measurements were carried out as described [65]. Values (n = 3) for XopL and each of its derivatives were compared to GFP (control) using unpaired Student's *t*-test.

### *In vitro* E3 ligase assays

*In vitro* E3 ligase assays were performed as described [17], [19]. *Arabidopsis* E2s used in this study were amplified from the CD4-16 cDNA library from the Arabidopsis Biological Resource Centre (ABRC, www.arabidopsis.org/abrc) and cloned into expression plasmid p15Tv-L (gi |134105575|). Plasmids encoding AtUBC28-variants R5A, F62A, K63A and A96D were generated using the Quick Change Site-Directed Mutagenesis II kit (Agilent Technologies Canada, Inc., Mississauga, Canada). The E1-enzyme, ubiquitin and ubiquitin mutants were purchased from Boston Biochem (Cambridge, USA). Ubiquitin- and His antibodies were purchased from EMD Millipore (Billerica, USA) and Qiagen (Toronto, Canada), respectively. His-tagged UBE2D2 was prepared as described [19], and *Arabidopsis* wild-type and mutant His-tagged E2s were purified accordingly. Sequences of oligonucleotides are available upon request.

*In vitro* ubiquitination reactions were analyzed by LC-MS/MS on OrbitrapVelos as described [66]. Briefly, 20 µl reactions containing 0.029 µM E1, 3 µM E2, 6 µM E3, 25 µM ubiquitin and 10 mM ATP (in 50 mM Tris pH 7.5 buffer, with 0.1 M NaCl, 10 mM MgCl_2_ and 0.5 mM DTT) were incubated at 25°C for 3 hours. Reactions were stopped by the addition of 2× Laemmli buffer and incubation for 5 minutes at 95°C. Proteins were separated by SDS-PAGE, and the gel band corresponding to >100 kDa excised and trypsinized. 1/10th of each band was analyzed in duplicates.

### Protein purification, expression and crystallization

Fragments of *Xcv*_XopL_ (XCV3220, gi 28872465) and *Xanthomonas campestris* pv. *campestris* str. ATCC 33913 XopL (XCC4186, gi 21233603) were cloned into expression plasmid p15Tv-L, followed by transformation of *E. coli* BL21(DE3)-RIPL (Agilent Technologies Canada, Inc., Mississauga, Canada). After optimizing solubility, *E. coli* cells expressing XopL fragments were cultured in 1 l LB at 37°C to an optical density (600 nm) of approximately 1.2, before IPTG was added to induce protein expression. Selenomethionine-enriched protein was produced following growth and induction of cells in SeMet high-yield media (Shanghai Medicilon, Shanghai, China). After induction, bacteria were incubated overnight on a shaker at 25°C. Cells were harvested by centrifugation, disrupted by sonication, and the insoluble material was removed by centrifugation. XopL fragments were purified using Ni-NTA affinity chromatography and dialyzed at 4°C in 10 mM HEPES (pH 7.5), 500 mM NaCl and 0.5 mM TCEP, concentrated to >15 mg/ml and stored at −70° C.

Crystallization trials were performed at room temperature using hanging-drop vapor diffusion with an optimized sparse matrix crystallization screen [67], with or without limiting amounts of proteases [68] including TEV. XopL[aa 144–450] crystals were grown at 25 mg/ml. The XopL[aa 144–450] crystal used for data collection (see Table 1) was grown from a crystallization liquor containing 0.2 M Potassium Sulfate and 20% PEG3350 monodisperse (Hampton Research, Aliso Viejo, USA) and cryoprotected in a similar buffer containing 10% glycerol and flash-frozen in liquid nitrogen, while the XopL[aa 474–660] crystal was grown using a protein concentration of 26 mg/ml from a crystallization liquor containing 0.1 M Tris pH 8.5, 0.2 M Sodium Acetate, 30% PEG4K and 4% ethylene glycol, cryoprotected using Paratone-N oil (Hampton Research) and flash-frozen in liquid nitrogen.

### Data collection, structure determination and refinement

The structure of XopL[aa 144–450] was determined by a crystal derived from selenomethionine-enriched protein with SAD phasing using a peak wavelength of λ = 0.97937 Å. Diffraction data were collected at 100° K at APS beamline 19-BM. Diffraction data were integrated and scaled at the beamline using HKL3000 [69]. Positions of heavy atoms were found using SHELXD [70], followed by solvent flattening using SHELXE [71], which was in turn used to automatically build an initial model using ArpWARP [72], all used within the CCP4 program suite [73]. The model was improved by alternate cycles of manual building and water-picking using COOT [74] and restrained refinement against a maximum-likelihood target with 5% of the reflections randomly excluded as an *R*_free_ test set. These refinement steps were performed using REFMAC in the CCP4 program suite. In addition we refined using Phenix.refine from the PHENIX crystallography suite [75], [76]. The final model contained a nearly complete chain containing 4 residues in the Ni-affinity tag and residues 145–437, in which the C-terminal Gly residue from the tag, residues 144, 297 and 438–450 were omitted due to protein disorder, and was refined to an *R*_work_ and *R*_free_ of 17.1 and 22.6%, respectively, including TLS parameterization [77], [78]. The structure of XopL[aa 474–660] was also solved by SAD phasing at peak wavelength (λ = 0.97921 Å) using a selenomethionine-enriched crystal. Structure solution, model building and refinement followed a similar protocol as for XopL[aa 144–450]. However, during refinement, phenix.xtriage, as part of the PHENIX crystallography suite, we detected merohedral twinning with twin law h, -h-k, -l and a twinning fraction of 0.273. Refinement then proceeded with a newly derived *R*_free_ set to take the twinning into consideration. As stated above, the final model contained three molecules in the asymmetric unit. Molecule A contains a complete chain involving the 5 most C-terminal residues from the Ni-affinity tag followed by residues 474–639. No electron density for residues 641–660 was observed due to protein disorder. Molecules B and C contained a very similar chain. In addition, in molecule B, the 6 most C-terminal residues of the Ni-affinity tag were modeled as well as residues 640–642. In molecule C, residues 474–476 were not modeled due to protein disorder, but residue 640 was. The final model (to 1.8 Å) was refined to an *R*_work_ and *R*_free_ of 15.2 and 19.4%, respectively.

Data collection, phasing and structure refinement statistics for both structures are summarized in Table 1. The Ramachandran plot generated by PROCHECK [79] showed very good stereochemistry overall with 99.6 and 100% of the residues in the most favored and additional allowed regions for XopL[aa 144–450] and XopL[aa 474–660], respectively.

### Mesophyll protoplast transient expression assay and immunoblot-based detection of MAPK activity

Transient expression experiments with *A. thaliana* (Col-0)-derived protoplasts were carried out as described [28]. Protoplast samples were co-transformed with the *NHL10* promoter-luciferase construct [27], [28], *pUBQ10-GUS* [80] and either *p35S*-effector gene constructs (*xopL*, *xopL_Q612A_*, *xopL_CTD_[aa450–660]*) or *p35S-cfp* as control (10 µg total DNA per 100 µl protoplasts; ratio 1∶1∶1). Activity of MAPKs was determined by protein extraction and immunoblotting using a specific pTepY-antibody as described previously [25]. GUS-activity was determined by measuring the turnover of 4-MUG (4-Methylumbelliferyl-β-D-glucuronide) with a Cytofluor II Platereader (Millipore Corp.; excitation 380 nm, emission 460 nm).

### Accession numbers

Coordinates for the XopL LRR domain (XopL[aa 144–450]) and the C-terminal domain (XopL[aa 474–660]) structures were deposited at the Protein Data Bank with accession codes 4FCG and 4FC9, respectively. XCV3220 (XopL) and XCC4186 (XccXopL) are targets APC108260 and APC105826 of the Midwest Center for Structural Genomics, respectively.

## Supporting Information

Figure S1 **Multiple sequence alignment of XopL homologues.** The amino acid sequences of XopL from *Xcv* and homologous proteins from other *Xanthomonas* spp. were aligned by ClustalX [12]. Red cylinders, blue arrows, black lines and dashed black lines represent helical, β-strand, structured loop and disordered regions in XopL, respectively as observed in the XopL[aa 144–450] and XopL[aa 474–660] structures. Cyan lines represent the ordered vector sequences observed in both the XopL[aa 144–450] and XopL[aa 474–660] structures. Mutated residues in the C-terminal domain of XopL, which abrogated PCD are marked with magenta circles or boxes. Mutated residues which elicited cell death similar to wild-type XopL are labeled with blue circles. Secondary structural elements are labeled, but helical regions <5 residues are marked and not labeled, as they may be considered helical loops rather than helices per se. Sequences of XopL and homologous proteins were aligned in the following order: XopL, *X. campestris* pv. *vesicatoria* 85-10 (Xcv), gi|78048776|; PopC, *X. oryzae* pv. *oryzicola* (X. oryzicola), gi|108946646|; PXO016102, *X. oryzae* pv. *oryzae* PXO99A (Xoo_PXO99A), gi|188577374|; XopL, *X. perforans* 91-118 (X. perforans), gi|325925746|; XAC3090, *X. axonopodis* pv. *citri* 306 (Xac_306), gi|77748695|; XopL, *X. fuscans* spp. *aurantifolii* ICPB 11122 (X. fuscans aurant), gi|294627335|;XopL, *X. gardneri* ATCC 19865 (X. gardneri), gi|325919350|; and XCC4186, *X. campestris* pv. *campestris* ATCC 33913 (Xcc), gi|21233603|.
(TIF)

Figure S2 **Genetic analysis of the type III effector candidate XopL.** (**A**) RT-PCR analysis of the effector gene *xopL*. Fragments were amplified from cDNA derived from *Xcv* strains 85-10, 85* and 85*Δ*hrpX* using specific primers. Genomic DNA, H_2_O and 16S rRNA were used as controls. (**B**) Type III secretion assay using the XopL_1–92_-AvrBs3Δ2 reporter fusion. Strains 85* (wt) and 85*Δ*hrcV* (Δ*hrcV*) expressing *xopL_1–92_-avrBs3Δ2* were grown in T3 secretion-inducing medium. Total cell extracts (TE) and culture supernatants (SN) were analyzed by immunoblotting using an AvrBs3-specific antibody. (**C**) *Xcv* strains described in (B), 85-10 and 85*Δ*hpaB* were tested for translocation of XopL_1–92_-AvrBs3Δ2 in AvrBs3-responsive pepper plants (ECW-30R). Leaves were harvested 4 dpi and bleached in ethanol for better visualization of the hypersensitive response (HR). (**D**) Leaves of susceptible (ECW) and resistant (ECW-10R) pepper plants were inoculated with *Xcv* wild-type strain 85-10 (wt) and a genomic deletion mutant of *xopL* (Δ*xopL*) at 10^8^ cfu/ml. Pictures of disease symptoms (ECW) were taken 6 dpi. For better visualization of the HR, leaves were bleached in ethanol 2 dpi.
(TIF)

Figure S3 **Expression of XopL-HA in protoplasts.** (**A**) Total protein extracted from protoplasts described in Figure 2D were subjected to an anti-HA immunoblot to detect expression of CFP, AvrPto, XopL, XopL_Q612A_, XopL_LRR_ and XopL_CTD_. (**B**) To determine viability of the protoplasts, GUS (β-glucuronidase) measurements were carried out at the end of the experiment as explained in Figure 2B. There is no statistically significant difference between the samples (1way ANOVA with Kruskal-Wallis post test; n = 9).
(TIF)

Figure S4 **SDS-PAGE of XopL fragments used in this study following protein purification.** Note that a persistent contaminant in purified full-length XopL is denoted by an asterisk.
(TIF)

Figure S5 **Analysis of cell death induction and ubiquitination by XopL and different derivatives in** ***Nicotiana benthamiana*** **.** *Agrobacterium*-mediated expression of *gfp*, *xopL* and constructs encoding the following XopL mutant derivatives: Δ163–185, Δ330–336, D502A, R505A N506A, A512E P513A, K578A, A579W, P517A K519A R520A, H584A L585A G586E, E598A S600A, L619A, XopL[aa 1–449] (LRR), XopL[aa 450–660] (CTD) in leaves of *N. benthamiana* at 8×10^8^ cfu/ml. (**A**) Phenotypes of the inoculated leaf area were documented 6 dpi. (**B**) Electrolyte leakage measurements for quantification of cell death reactions 2 dpi (light grey bars) and 4 dpi (dark grey bars), respectively. Bars represent triplicates of 5 leaf discs each and standard deviations thereof. Asterisks indicate statistically significant differences compared to GFP control (*t*-test, *P*<0.05). (**C**) Leaf tissue was harvested 2 dpi and plant protein extracts were analyzed by immunoblotting using *Strep*-tag- (α-Strep) and ubiquitin-specific antibodies (α-Ub), respectively. Signals specific for full length XopL, XopL[aa 1–449] and XopL[aa 450–660] are labeled. (Ub)_n_ indicates polyubiqutination. Equal loading is demonstrated by Ponceau staining of Rubisco. The experiments were performed three times with similar results.
(TIF)

Figure S6 ***In vitro*** **E3 ligase reaction of the XL-box and various point mutants.** (**A**) Ubiquitination reaction of the wild-type and mutated XL-box fragments. As denoted, ubiquitination reactions were performed for 2 hours, run on a 10–15% SDS-PAGE step gradient gel and probed with anti-ubiquitin antibodies (α-Ub). To demonstrate similar loading, a 15% SDS-PAGE gel was run of the starting material (t = 0) and both stained with Coomassie blue (**B**) or probed with anti-His antibodies (α-His) (**C**).
(TIF)

Text S1 This file includes Supplemental Tables S1, S2, S3 and Supplemental References.
(DOC)
