# Correction: A Pathogen Type III Effector with a Novel E3 Ubiquitin Ligase Architecture

**DOI:** 10.1371/annotation/8c6eaae4-72a7-460a-8b1a-f855731f3706

**Published:** 2013-05-07

**Authors:** Alexander U. Singer, Sebastian Schulze, Tatiana Skarina, Xiaohui Xu, Hong Cui, Lennart Eschen-Lippold, Monique Egler, Tharan Srikumar, Brian Raught, Justin Lee, Dierk Scheel, Alexei Savchenko, Ulla Bonas

There is an error in the funding statement. The number of the National Institutes of Health grant "GM074942" should instead be "GM094585".

