# Supplementary figures and images for: A Pathogen Type III Effector with a Novel E3 Ubiquitin Ligase Architecture
Source: PLoS Pathog. 2013 Jan 24;9(1):e1003121. doi: 10.1371/journal.ppat.1003121 (PMC3554608; doi:10.1371/journal.ppat.1003121)

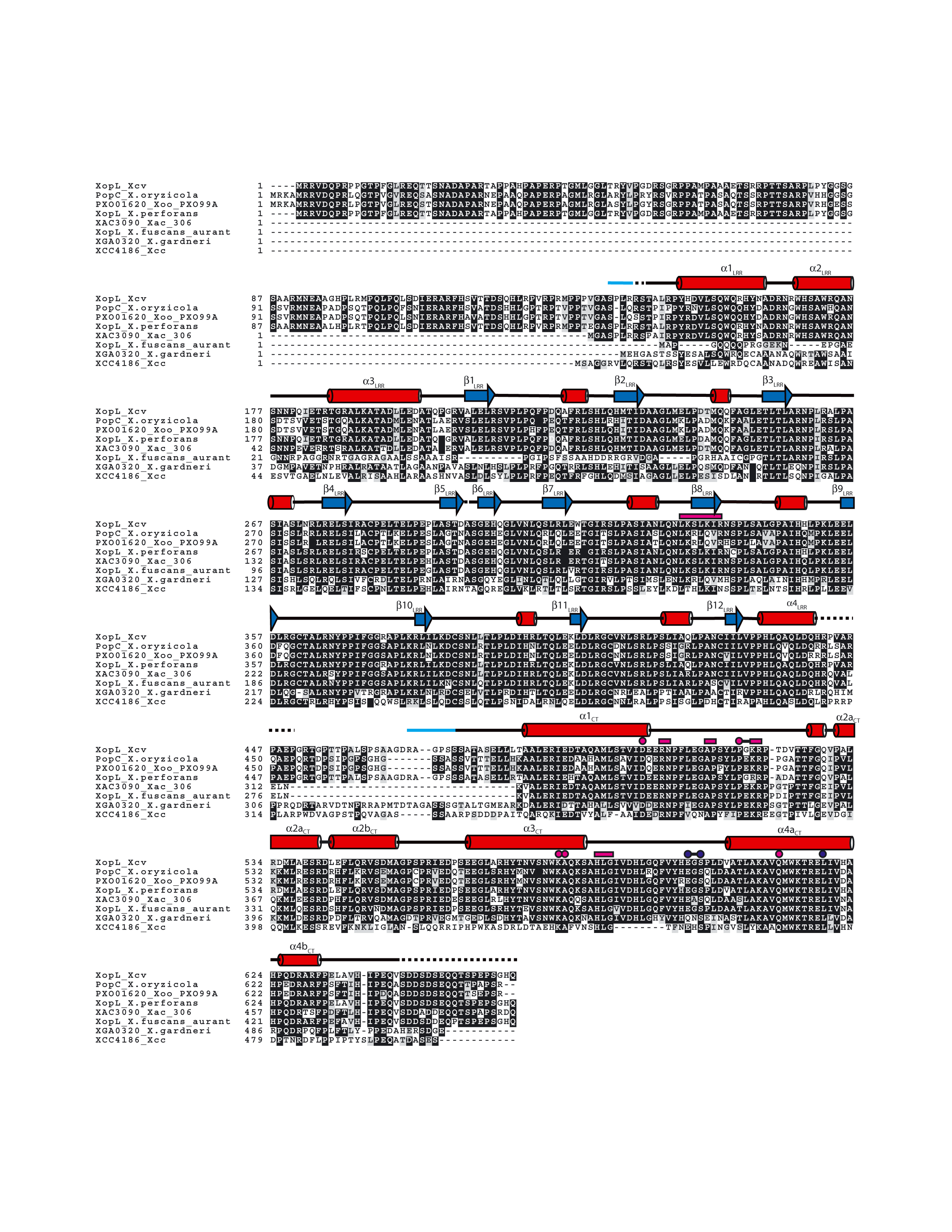

Supplement: Figure S1 — Multiple sequence alignment of XopL homologues. The amino acid sequences of XopL from Xcv and homologous proteins from other Xanthomonas spp. were aligned by ClustalX [12]. Red cylinders, blue arrows, black lines and dashed black lines represent helical, β-strand, structured loop and disordered regions in XopL, respectively as observed in the XopL[aa 144–450] and XopL[aa 474–660] structures. Cyan lines represent the ordered vector sequences observed in both the XopL[aa 144–450] and XopL[aa 474–660] structures. Mutated residues in the C-terminal domain of XopL, which abrogated PCD are marked with magenta circles or boxes. Mutated residues which elicited cell death similar to wild-type XopL are labeled with blue circles. Secondary structural elements are labeled, but helical regions <5 residues are marked and not labeled, as they may be considered helical loops rather than helices per se. Sequences of XopL and homologous proteins were aligned in the following order: XopL, X. campestris pv. vesicatoria 85-10 (Xcv), gi|78048776|; PopC, X. oryzae pv. oryzicola (X. oryzicola), gi|108946646|; PXO016102, X. oryzae pv. oryzae PXO99A (Xoo_PXO99A), gi|188577374|; XopL, X. perforans 91-118 (X. perforans), gi|325925746|; XAC3090, X. axonopodis pv. citri 306 (Xac_306), gi|77748695|; XopL, X. fuscans spp. aurantifolii ICPB 11122 (X. fuscans aurant), gi|294627335|;XopL, X. gardneri ATCC 19865 (X. gardneri), gi|325919350|; and XCC4186, X. campestris pv. campestris ATCC 33913 (Xcc), gi|21233603|. (TIF) [file ppat.1003121.s001.tif]

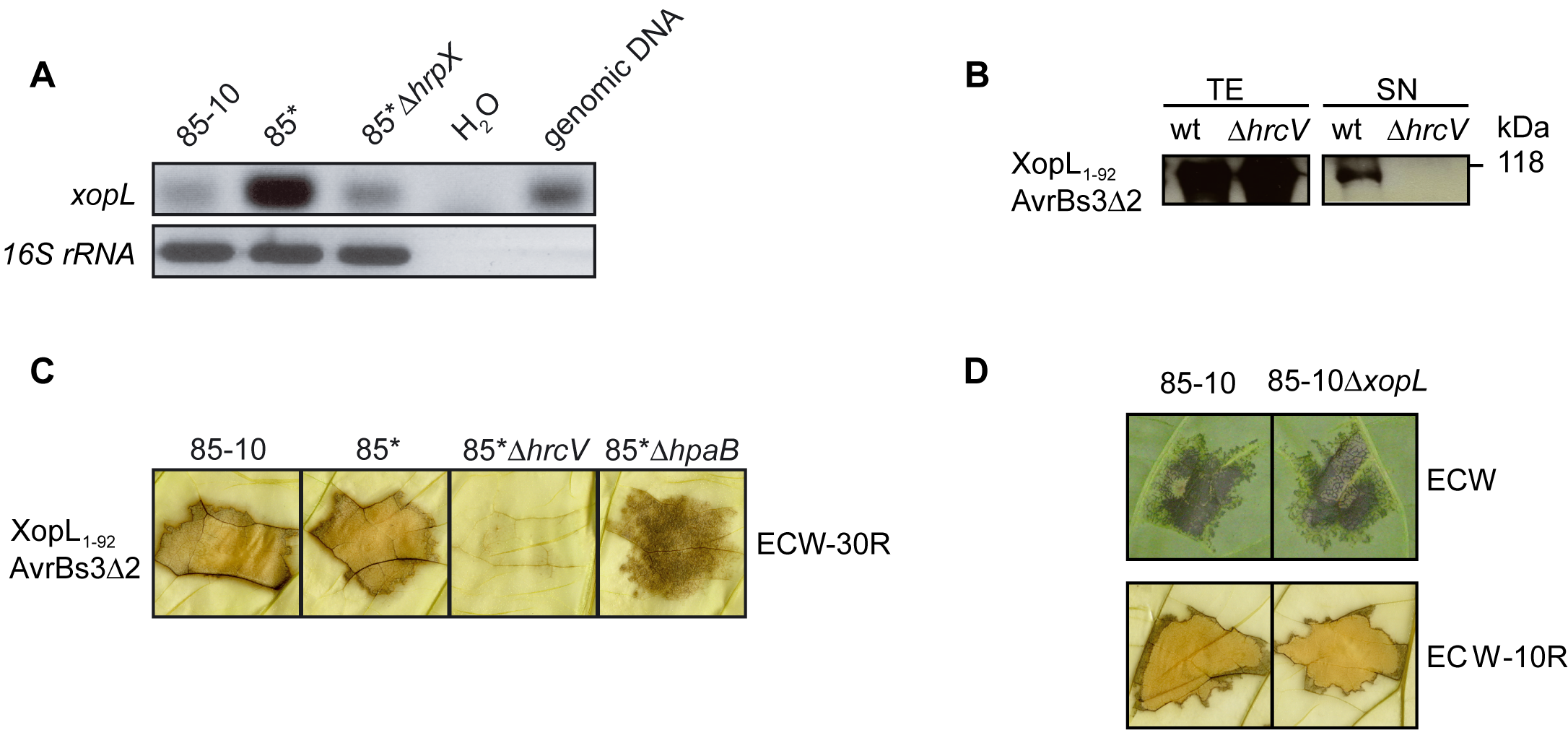

Supplement: Figure S2 — Genetic analysis of the type III effector candidate XopL. (A) RT-PCR analysis of the effector gene xopL. Fragments were amplified from cDNA derived from Xcv strains 85-10, 85* and 85*ΔhrpX using specific primers. Genomic DNA, H2O and 16S rRNA were used as controls. (B) Type III secretion assay using the XopL1–92-AvrBs3Δ2 reporter fusion. Strains 85* (wt) and 85*ΔhrcV (ΔhrcV) expressing xopL1–92-avrBs3Δ2 were grown in T3 secretion-inducing medium. Total cell extracts (TE) and culture supernatants (SN) were analyzed by immunoblotting using an AvrBs3-specific antibody. (C) Xcv strains described in (B), 85-10 and 85*ΔhpaB were tested for translocation of XopL1–92-AvrBs3Δ2 in AvrBs3-responsive pepper plants (ECW-30R). Leaves were harvested 4 dpi and bleached in ethanol for better visualization of the hypersensitive response (HR). (D) Leaves of susceptible (ECW) and resistant (ECW-10R) pepper plants were inoculated with Xcv wild-type strain 85-10 (wt) and a genomic deletion mutant of xopL (ΔxopL) at 108 cfu/ml. Pictures of disease symptoms (ECW) were taken 6 dpi. For better visualization of the HR, leaves were bleached in ethanol 2 dpi. (TIF) [file ppat.1003121.s002.tif]

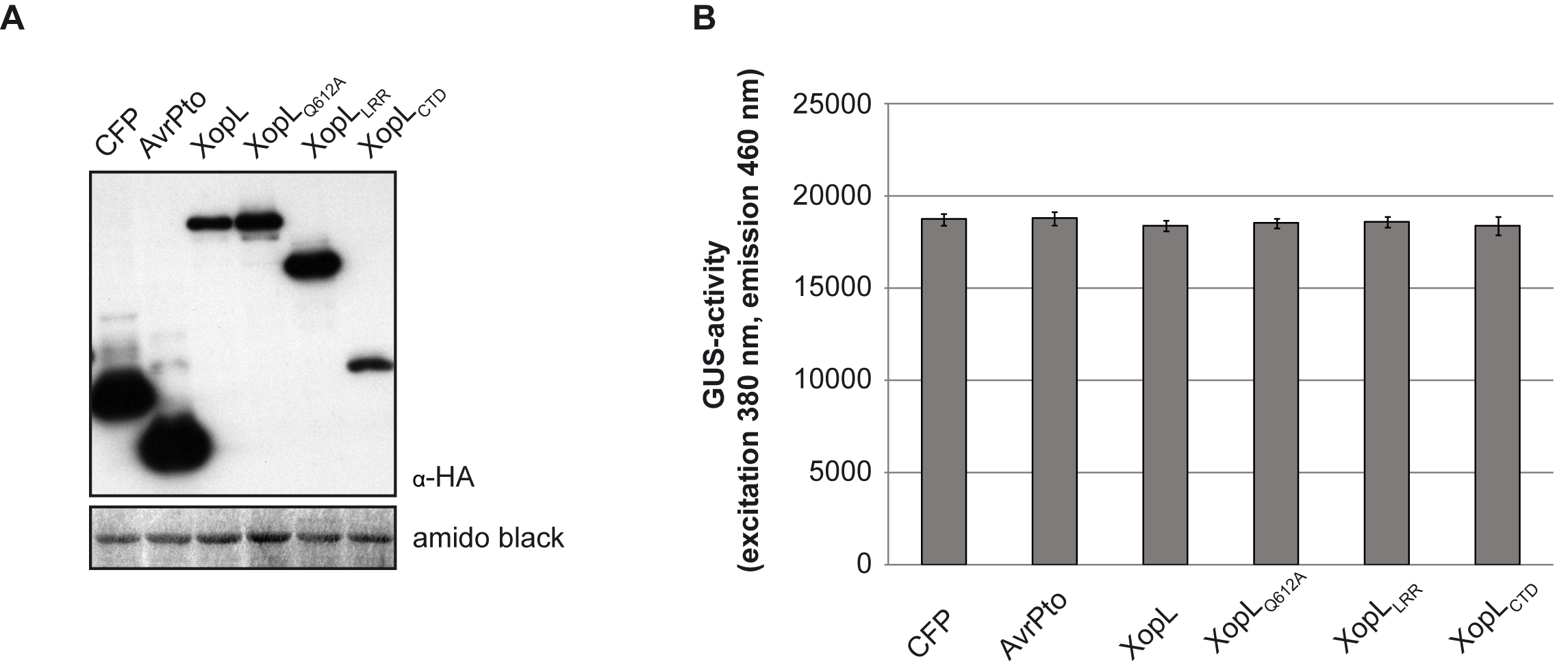

Supplement: Figure S3 — Expression of XopL-HA in protoplasts. (A) Total protein extracted from protoplasts described in Figure 2D were subjected to an anti-HA immunoblot to detect expression of CFP, AvrPto, XopL, XopLQ612A, XopLLRR and XopLCTD. (B) To determine viability of the protoplasts, GUS (β-glucuronidase) measurements were carried out at the end of the experiment as explained in Figure 2B. There is no statistically significant difference between the samples (1way ANOVA with Kruskal-Wallis post test; n = 9). (TIF) [file ppat.1003121.s003.tif]

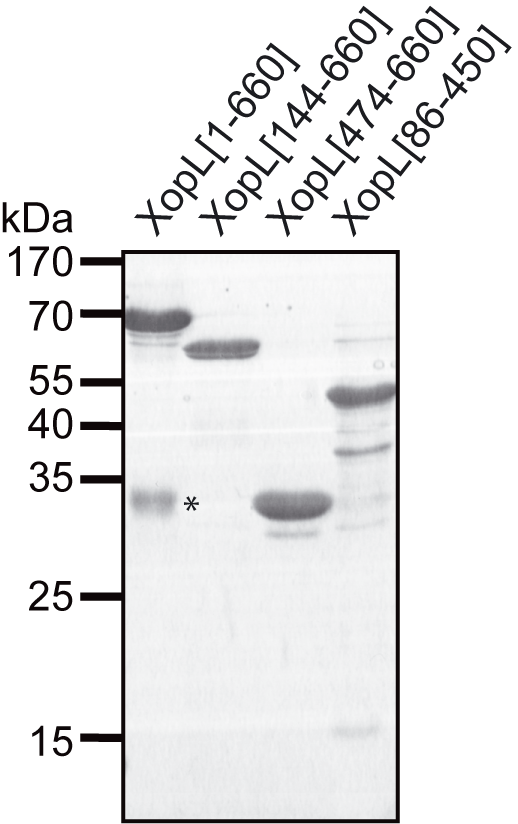

Supplement: Figure S4 — SDS-PAGE of XopL fragments used in this study following protein purification. Note that a persistent contaminant in purified full-length XopL is denoted by an asterisk. (TIF) [file ppat.1003121.s004.tif]

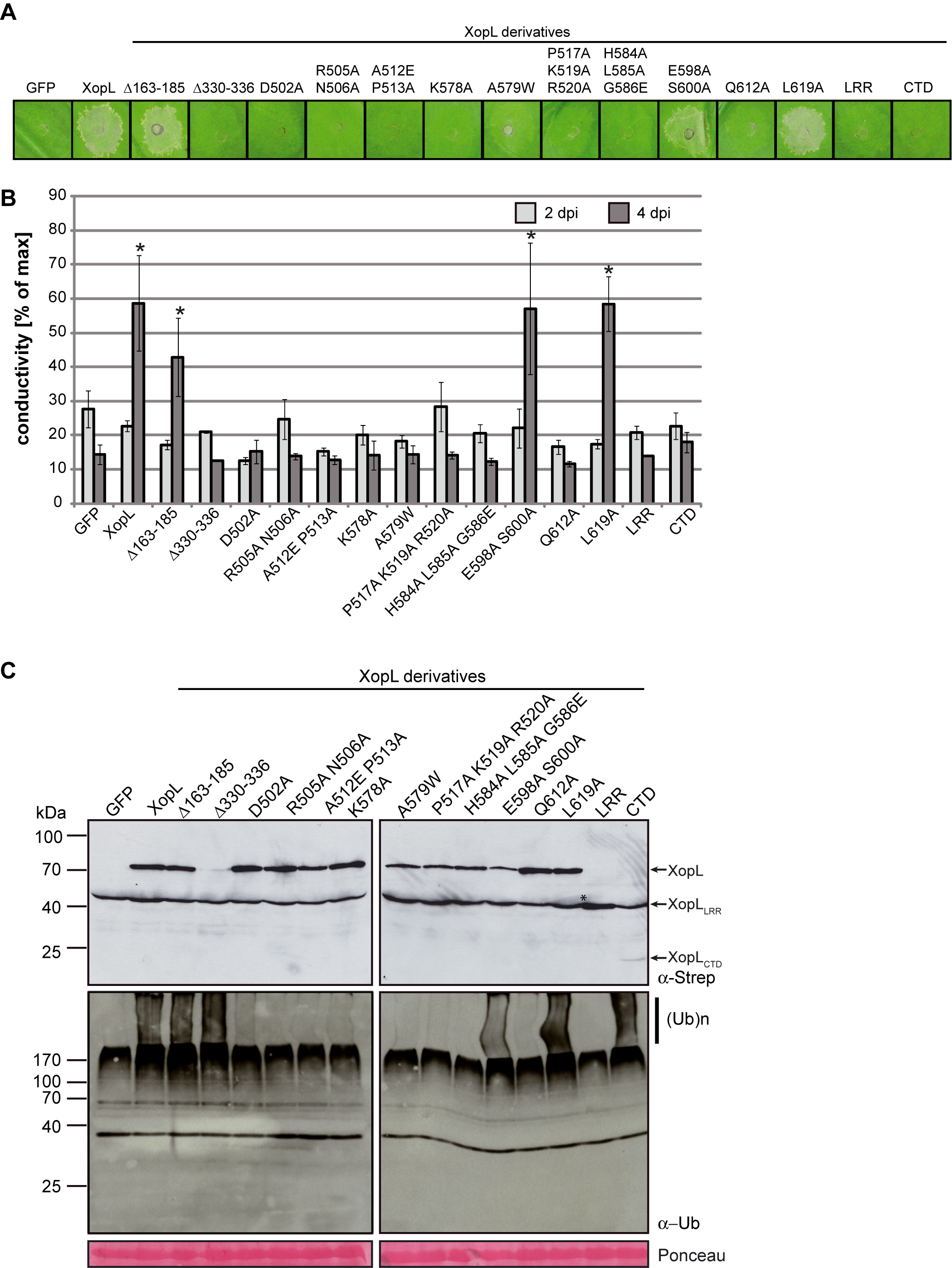

Supplement: Figure S5 — Analysis of cell death induction and ubiquitination by XopL and different derivatives in Nicotiana benthamiana . Agrobacterium-mediated expression of gfp, xopL and constructs encoding the following XopL mutant derivatives: Δ163–185, Δ330–336, D502A, R505A N506A, A512E P513A, K578A, A579W, P517A K519A R520A, H584A L585A G586E, E598A S600A, L619A, XopL[aa 1–449] (LRR), XopL[aa 450–660] (CTD) in leaves of N. benthamiana at 8×10^8^ cfu/ml. (A) Phenotypes of the inoculated leaf area were documented 6 dpi. (B) Electrolyte leakage measurements for quantification of cell death reactions 2 dpi (light grey bars) and 4 dpi (dark grey bars), respectively. Bars represent triplicates of 5 leaf discs each and standard deviations thereof. Asterisks indicate statistically significant differences compared to GFP control (t-test, P<0.05). (C) Leaf tissue was harvested 2 dpi and plant protein extracts were analyzed by immunoblotting using Strep-tag- (α-Strep) and ubiquitin-specific antibodies (α-Ub), respectively. Signals specific for full length XopL, XopL[aa 1–449] and XopL[aa 450–660] are labeled. (Ub)n indicates polyubiqutination. Equal loading is demonstrated by Ponceau staining of Rubisco. The experiments were performed three times with similar results. (TIF) [file ppat.1003121.s005.tif]

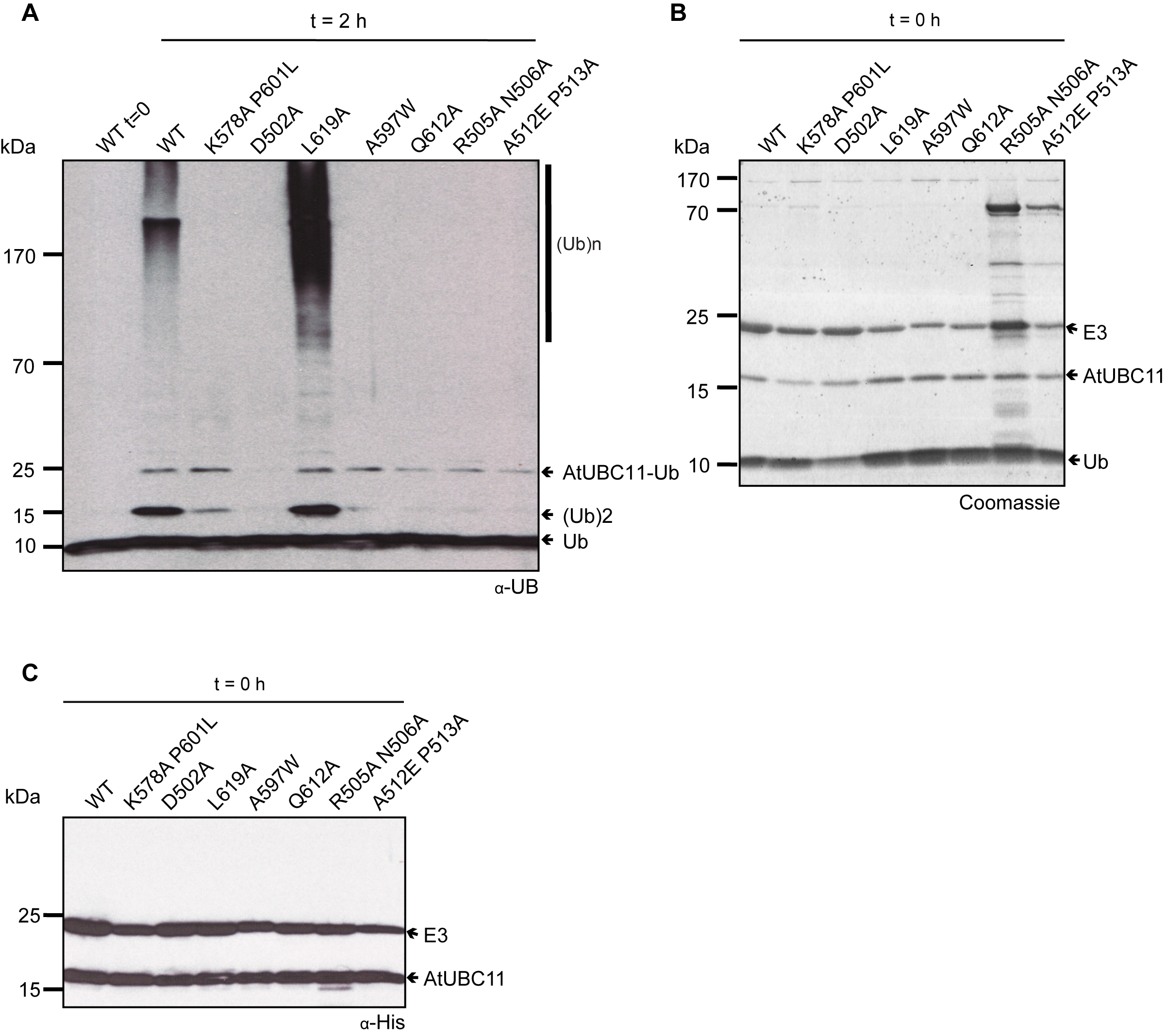

Supplement: Figure S6 — In vitro E3 ligase reaction of the XL-box and various point mutants. (A) Ubiquitination reaction of the wild-type and mutated XL-box fragments. As denoted, ubiquitination reactions were performed for 2 hours, run on a 10–15% SDS-PAGE step gradient gel and probed with anti-ubiquitin antibodies (α-Ub). To demonstrate similar loading, a 15% SDS-PAGE gel was run of the starting material (t = 0) and both stained with Coomassie blue (B) or probed with anti-His antibodies (α-His) (C). (TIF) [file ppat.1003121.s006.tif]
