# Supplementary material for: A Pathogen Type III Effector with a Novel E3 Ubiquitin Ligase Architecture
Source: PLoS Pathog. 2013 Jan 24;9(1):e1003121. doi: 10.1371/journal.ppat.1003121 (PMC3554608; doi:10.1371/journal.ppat.1003121)
Supplement: Text S1 — This file includes Supplemental Tables S1, S2, S3 and Supplemental References. (DOC) [file ppat.1003121.s007.doc]

**Supplemental Text S1**

**Type III secretion and translocation assays of XopL**

The T3S system of *Xcv* is encoded by the 23 kb chromosomal *hrp* (hypersensitive response and pathogenicity) gene cluster, which is essential for bacterial growth and disease symptoms on susceptible plants as well as for induction of the HR in resistant host and nonhost plants [1]. Expression of *hrp* genes is induced *in planta* by the OmpR-family regulator HrpG which controls the expression of a genome-wide regulon [2] including the AraC-type transcriptional activator *hrpX* [3,4]. HrpX binds to *cis*-regulatory PIP (plant-inducible promoter) boxes in the promoter regions of *hrp* and other genes that contribute to virulence [5].

To analyze type III-dependent secretion and translocation of XopL, a 1269 bp fragment containing the native promoter and 276 bp 5’ coding sequence (92 aa) was amplified from genomic DNA of *Xcv* 85-10, cloned into the effector reporter plasmid pL6GW356 [6] and conjugated into *Xcv* strain 85-10 and derivatives thereof. Strain 85* is a derivative of strain 85-10 and expresses a constitutively active HrpG protein resulting in constitutive expression of the T3S system. The T3S mutant 85*∆*hrcV* features the deletion of a gene encoding a conserved inner membrane component of the T3S system [7], and 85*∆*hpaB* lacks the general T3S-chaperone, which is important for secretion and translocation of certain effectors [8,9]. The native promoter and 5´ coding region of *xopL* was fused to the reporter construct *avrBs3∆2*, which lacks a T3S signal but retains a functional effector domain [6,10].

*In vitro* secretion of XopL_1-92_AvrBs3∆2 clearly depended on a functional T3S system, because XopL_1-92_-AvrBs3∆2 was detectable in culture supernatants of strain 85* but not in the T3S-deficient strain 85^*^∆*hrcV* (Figure S2B). HrcJ, a lipoprotein in the inner membrane of the T3S system [7], was used as lysis control (data not shown). Translocation was tested by inoculation of strains 85-10, 85*, 85*∆*hrcV* and 85*∆*hpaB*, all carrying the XopL_1-92_-AvrBs3∆2 fusion construct, into leaves of pepper ECW-30R plants, that carry the *Bs3*-resistance gene. As shown in Figure S2C, 85-10(XopL_1-92_-AvrBs3∆2) induced the HR. This indicates that *xopL* is expressed during infection of pepper leading to expression and translocation of XopL_1-92_-AvrBs3∆2. 85* expressing XopL_1-92_-AvrBs3∆2 also triggered the HR. As expected, no HR was induced by strain 85*∆*hrcV* expressing XopL_1-92_-AvrBs3∆2 (Figure S2C). However, XopL_1-92_-AvrBs3∆2 was also translocated by the ∆*hpaB* strain lacking the T3S chaperone, which is in contrast to the XopL homologue XC_4273 from *X. c.* pv. *campestris* [11].

Table S1. MS/MS analysis of XopL ubiquitination reactions.

|  |  |  |  | Spectral Counts | | | | | |  | |
| --- | --- | --- | --- | --- | --- | --- | --- | --- | --- | --- | --- |
| *Xanthomonas* XopL-derivative (E3 Enzyme) | E2 Enzyme | MS Run | Total Ub  Peptides | K6 | K11 | K27 | K29 | K33 | K48 | K63 | Total Linkages |
| Xcv3220_144-660 | ATUBC28 | 1 | 2712 | 0 | 482 | 0 | 0 | 145 | 54 | 228 | 909 |
| Xcv3220_144-660 | ATUBC28 | 2 | 3700 | 0 | 654 | 0 | 0 | 203 | 58 | 365 | 1280 |
| Xcv3220_144-660 | hUBE2D2 | 1 | 389 | 0 | 19 | 0 | 0 | 5 | 3 | 3 | 30 |
| Xcv3220_144-660 | hUBE2D2 | 2 | 482 | 0 | 38 | 0 | 0 | 3 | 6 | 3 | 50 |
| Xcv3220_474-660 | hUBE2D2 | 1 | 431 | 0 | 18 | 0 | 0 | 6 | 12 | 3 | 39 |
| Xcv3220_474-660 | hUBE2D2 | 2 | 482 | 0 | 30 | 0 | 0 | 5 | 3 | 6 | 44 |
| Xcc4186_330-496 | ATUBC28 | 1 | 830 | 0 | 98 | 0 | 0 | 0 | 3 | 3 | 104 |
| Xcc4186_330-496 | ATUBC28 | 2 | 850 | 0 | 108 | 0 | 0 | 0 | 3 | 3 | 114 |
| Xcv3220_144-660 | ATUBC11 | 1* | 16 | 0 | 0 | 0 | 0 | 0 | 0 | 0 | 0 |
| Xcv3220_144-660 | ATUBC11 | 2* | 10 | 0 | 0 | 0 | 0 | 0 | 0 | 0 | 0 |
| Xcv3220_144-660 | ATUBC11 | 1 | 632 | 0 | 48 | 0 | 0 | 48 | 17 | 0 | 113 |
| Xcv3220_144-660 | ATUBC11 | 2 | 649 | 0 | 54 | 0 | 0 | 50 | 15 | 0 | 119 |

|  |  |  |  | Normalized Linkage Percentage | | | | | | |
| --- | --- | --- | --- | --- | --- | --- | --- | --- | --- | --- |
| *Xanthomonas* XopL-derivative (E3 Enzyme) | E2 Enzyme | MS Run | Total Ub  Peptides | K6 | K11 | K27 | K29 | K33 | K48 | K63 |
| Xcv3220_144-660 | ATUBC28 | 1 | 2712 | 0% | 57% | 0% | 0% | 16% | 5% | 22% |
| Xcv3220_144-660 | ATUBC28 | 2 | 3700 | 0% | 55% | 0% | 0% | 16% | 4% | 25% |
| Xcv3220_144-660 | hUBE2D2 | 1 | 389 | 0% | 66% | 0% | 0% | 17% | 9% | 8% |
| Xcv3220_144-660 | hUBE2D2 | 2 | 482 | 0% | 79% | 0% | 0% | 6% | 10% | 5% |
| Xcv3220_474-660 | hUBE2D2 | 1 | 431 | 0% | 50% | 0% | 0% | 16% | 27% | 7% |
| Xcv3220_474-660 | hUBE2D2 | 2 | 482 | 0% | 71% | 0% | 0% | 11% | 6% | 12% |
| Xcc4186_330-496 | ATUBC28 | 1 | 830 | 0% | 95% | 0% | 0% | 0% | 2% | 2% |
| Xcc4186_330-496 | ATUBC28 | 2 | 850 | 0% | 96% | 0% | 0% | 0% | 2% | 2% |
| Xcv3220_144_660 | ATUBC11 | 1 | 632 | 0% | 44% | 0% | 0% | 43% | 13% | 0% |
| Xcv3220_144_660 | ATUBC11 | 2 | 649 | 0% | 47% | 0% | 0% | 42% | 11% | 0% |

Spectral counts for each ubiquitin (Ub) linkage type (along with unmodified Ub peptides) identified using the spectral matching algorithm SpectraST [13], with a ubiquitin spectral library ([14]; version Ub_Ubl_v5 (available at raughtlab.ca/resources/msresources.php)) in *in vitro* ubiquitination assays using *Xanthomonas* XopL-derivatives (E3), Arabidopsis E2 (ATUBC11 or ATUBC28) or human E2 (hUBE2D2), E1 and ubiquitin. *In vitro* reactions were incubated for 3 hours, except for XopL[aa 144-660] (Xcv3220_144_660) plus ATUBC11, which was stopped immediately at t=0 hours (denoted by a *) or incubated for 2 hours. Reactions were separated via SDS-PAGE, and products migrating at >100 kDa were subjected to in-gel digestion. The resulting peptides were identified using tandem mass spectrometry. Normalized percentages (%) [15] in each reaction are also provided.

Table S2. Arabidopsis E2 ubiquitin conjugating proteins used in E3 ligase assays.

| **Protein Name** | **Gene** | **GI** | **E2 family^1^** | **Human homologue** |
| --- | --- | --- | --- | --- |
| AtUBC11 | At3g08690 | 18398206 | VI | UBE2D2 |
| AtUBC13 | At3g46460 | 18408206 | V | UBE2G1 |
| AtUBC19 | At3g20060 | 18402475 | VIII | UBE2C |
| AtUBC28 | At1g64230 | 18408001 | VI | UBE2D2 |

^1^Based on the classification of Arabidopsis E2s in Kraft et al (2005).

**Table S3. XopL derivatives tested *in vivo* in this study^1^.**

| **#** | **Position of amino acid exchange or deletion** | **cell death^2^** | **E3-ligase-activity^3^** |
| --- | --- | --- | --- |
|  | none (XopL wild-type) | + | + |
| 1 | LRR-domain (∆450-660) | - | - |
| 2 | ∆163-185 | + | + |
| 3 | R258A | + | n.a. |
| 4 | R280A | + | n.a. |
| 5 | W314A | + | n.a. |
| 6 | R258A R280A | + | n.a. |
| 7 | R258A W314A | + | n.a. |
| 8 | R280A W314A | + | n.a. |
| 9 | R258A R280A W314A | + | n.a. |
| 10 | ∆330-336 | - | + |
| 11 | K334A | + | n.a. |
| 12 | R336A | + | n.a. |
| 13 | R359A | + | n.a. |
| 14 | K383A | + | n.a. |
| 15 | R359A R379A | + | n.a. |
| 16 | R359A K383A | + | n.a. |
| 17 | R379A K383A | + | n.a. |
| 18 | K403A | + | n.a. |
| 19 | C-terminal domain (∆1-449) | - | + |
| 20 | D502A | - | - |
| 21 | R505A N506A | - | - |
| 22 | A512E P513A | - | - |
| 23 | K578A | - | - |
| 24 | A579W | - | - |
| 25 | P517A K519A R520A | - | - |
| 26 | H584A L585A G586E | - | - |
| 27 | E598A S600A | + | + |
| 28 | Q612A | - | - |
| 29 | L619A | + | + |

^1^ XopL derivatives were analyzed by transient expression in *N. benthamiana* regarding their ^2^ability to trigger cell death and ^3^E3 ligase activity. (+): PCD or E3 ligase activity detectable. (-): PCD or E3 ligase activity not detectable, n.a. not analyzed)

**Supplemental References**

1. Bonas U, Schulte R, Fenselau S, Minsavage GV, Staskawicz BJ, et al. (1991) Isolation of a gene-cluster from *Xanthomonas campestris* pv. *vesicatoria* that determines pathogenicity and the hypersensitive response on pepper and tomato. Mol Plant-Microbe Interact 4: 81-88.

2. Noël L, Thieme F, Nennstiel D, Bonas U (2001) cDNA-AFLP analysis unravels a genome-wide *hrpG*-regulon in the plant pathogen *Xanthomonas campestris* pv. *vesicatoria*. Mol Microbiol 41: 1271-1281.

3. Wengelnik K, Bonas U (1996) HrpXv, an AraC-type regulator, activates expression of five of the six loci in the *hrp* cluster of *Xanthomonas campestris* pv. vesicatoria. J Bacteriol 178: 3462-3469.

4. Wengelnik K, Van den Ackerveken G, Bonas U (1996) HrpG, a key *hrp* regulatory protein of *Xanthomonas* *campestris* pv. *vesicatoria* is homologous to two-component response regulators. Mol Plant-Microbe Interact 9: 704-712.

5. Koebnik R, Krüger A, Thieme F, Urban A, Bonas U (2006) Specific binding of the *Xanthomonas campestris* pv. vesicatoria AraC-type transcriptional activator HrpX to plant-inducible promoter boxes. J Bacteriol 188: 7652-7660.

6. Noël L, Thieme F, Gabler J, Büttner D, Bonas U (2003) XopC and XopJ, two novel type III effector proteins from *Xanthomonas campestris* pv. vesicatoria. J Bacteriol 185: 7092-7102.

7. Rossier O, Van den Ackerveken G, Bonas U (2000) HrpB2 and HrpF from *Xanthomonas* are type III-secreted proteins and essential for pathogenicity and recognition by the host plant. Mol Microbiol 38: 828-838.

8. Büttner D, Lorenz C, Weber E, Bonas U (2006) Targeting of two effector protein classes to the type III secretion system by a HpaC- and HpaB-dependent protein complex from *Xanthomonas campestris* pv. *vesicatoria*. Mol Microbiol 59: 513-527.

9. Schulze S, Kay S, Büttner D, Egler M, Eschen-Lippold L, et al. (2012) Analysis of new type III effectors from *Xanthomonas* uncovers XopB and XopS as suppressors of plant immunity. New Phytol.

10. Szurek B, Rossier O, Hause G, Bonas U (2002) Type III-dependent translocation of the *Xanthomonas* AvrBs3 protein into the plant cell. Mol Microbiol 46: 13-23.

11. Jiang W, Jiang BL, Xu RQ, Huang JD, Wei HY, et al. (2009) Identification of six type III effector genes with the PIP box in *Xanthomonas campestris* pv. *campestris* and five of them contribute individually to full pathogenicity. Mol Plant Microbe Interact 22: 1401-1411.

12. Thompson JD, Gibson TJ, Plewniak F, Jeanmougin F, Higgins DG (1997) The CLUSTAL_X windows interface: flexible strategies for multiple sequence alignment aided by quality analysis tools. Nucleic Acids Res 25: 4876-4882.

13. Lam H, Deutsch EW, Eddes JS, Eng JK, Stein SE, et al. (2008) Building consensus spectral libraries for peptide identification in proteomics. Nat Methods 5: 873-875.

14. Srikumar T, Jeram SM, Lam H, Raught B (2010) A ubiquitin and ubiquitin-like protein spectral library. Proteomics 10: 337-342.

15. Sheng Y, Hong JH, Doherty R, Srikumar T, Shloush J, et al. (2012) A human ubiquitin conjugating enzyme (E2) - HECT E3 ligase structure-function screen. Mol Cell Proteomics.
